# A 25-gene classifier predicts overall survival in resectable pancreatic cancer

**DOI:** 10.1186/s12916-017-0936-z

**Published:** 2017-09-20

**Authors:** David J. Birnbaum, Pascal Finetti, Alexia Lopresti, Marine Gilabert, Flora Poizat, Jean-Luc Raoul, Jean-Robert Delpero, Vincent Moutardier, Daniel Birnbaum, Emilie Mamessier, François Bertucci

**Affiliations:** 1 Département d’Oncologie Moléculaire, Centre de Recherche en Cancérologie de Marseille, Inserm UMR1068, CNRS UMR725, Aix-Marseille Université, Marseille, France; 2 Département de Chirurgie Générale et Viscérale, AP-HM, Marseille, France; 3 Faculté de Médecine, Aix-Marseille Université, Marseille, France; 4 Département d’Oncologie Médicale, Institut Paoli-Calmettes, Marseille, France; 5 Département d’Anatomopathologie, Institut Paoli-Calmettes, Marseille, France; 6 Département d’Oncologie Chirurgicale, Institut Paoli-Calmettes, Marseille, France; 7 Département d’Oncologie Moléculaire, Institut Paoli-Calmettes, 232 Bd. Ste-Marguerite, 13009 Marseille, France

**Keywords:** Expression profiling, Pancreatic cancer, Prognosis, Survival

## Abstract

**Background:**

Pancreatic carcinoma is one of the most lethal human cancers. In patients with resectable tumors, surgery followed by adjuvant chemotherapy is the only curative treatment. However, the 5-year survival is 20%. Because of a strong metastatic propensity, neoadjuvant chemotherapy is being tested in randomized clinical trials. In this context, improving the selection of patients for immediate surgery or neoadjuvant chemotherapy is crucial, and high-throughput molecular analyses may help; the present study aims to address this.

**Methods:**

Clinicopathological and gene expression data of 695 pancreatic carcinoma samples were collected from nine datasets and supervised analysis was applied to search for a gene expression signature predictive for overall survival (OS) in the 601 informative operated patients. The signature was identified in a learning set of patients and tested for its robustness in a large independent validation set.

**Results:**

Supervised analysis identified 1400 genes differentially expressed between two selected patient groups in the learning set, namely 17 long-term survivors (LTS; ≥ 36 months after surgery) and 22 short-term survivors (STS; dead of disease between 2 and 6 months after surgery). From these, a 25-gene prognostic classifier was developed, which identified two classes (“STS-like” and “LTS-like”) in the independent validation set (*n* = 562), with a 25% (95% CI 18–33) and 48% (95% CI 42–54) 2-year OS (*P* = 4.33 × 10^–9^), respectively. Importantly, the prognostic value of this classifier was independent from both clinicopathological prognostic features and molecular subtypes in multivariate analysis, and existed in each of the nine datasets separately. The generation of 100,000 random gene signatures by a resampling scheme showed the non-random nature of our prognostic classifier.

**Conclusion:**

This study, the largest prognostic study of gene expression profiles in pancreatic carcinoma, reports a 25-gene signature associated with post-operative OS independently of classical factors and molecular subtypes. This classifier may help select patients with resectable disease for either immediate surgery (the LTS-like class) or neoadjuvant chemotherapy (the STS-like class). Its assessment in the current prospective trials of adjuvant and neoadjuvant chemotherapy trials is warranted, as well as the functional analysis of the classifier genes, which may provide new therapeutic targets.

**Electronic supplementary material:**

The online version of this article (doi:10.1186/s12916-017-0936-z) contains supplementary material, which is available to authorized users.

## Background

With a mortality rate close to the incidence rate (331,000 deaths worldwide for 338,000 new cases in 2012 [1]), pancreatic carcinoma is one of the most lethal human cancers. Advances in systemic chemotherapy and radiotherapy provided limited improvement in survival, and the 5-year overall survival (OS) remains close to 5%. Only 50% of newly diagnosed patients have a non-metastatic disease with either a resectable or borderline resectable tumor (20%) or an unresectable locally-advanced tumor (30%) [2]. In patients with a resectable tumor, complete surgical removal followed by adjuvant chemotherapy is the only curative treatment. However, most of the patients display distant relapse; the median OS remains 23 months on average, and the 5-year survival is 20%. The mortality of surgery has decreased during the last 30 years, but its morbidity remains at approximately 50% [3].

The high rates of patients with stage IV and experiencing distant relapses after surgery in non-metastatic stages along with preclinical data suggest that metastatic spread may precede local tumor formation [4]. This has led to the emerging consensus that pancreatic cancer is a systemic disease already at diagnosis. More effective systemic therapies should confer an increased likelihood of cure after resection. Neoadjuvant chemotherapy, standardly used for borderline resectable and unresectable locally advanced diseases [2], is being tested in resectable tumors with several objectives [5], including early treatment of occult micrometastases, avoidance of unnecessary and morbid resection for rapidly metastasizing tumors, improvement of the likelihood of margin-negative resection, and better chemotherapy delivery than in adjuvant settings when surgical complications may delay or worsen chemotherapy tolerability. Other advantages include the ability to assess tumor response and to search for biological predictors for pathological response, which is associated with survival [6, 7]. Neoadjuvant chemotherapy provided interesting results in resectable pancreatic cancer in a few institutional prospective phase II studies [8–10], and randomized phase II/III studies are ongoing [2]. However, this approach faces potential hurdles such as a possible missed opportunity for curative surgery and the absence of surgical staging. In this context, improving our ability to select patients for either immediate surgery or neoadjuvant chemotherapy is crucial, and represents an area of high need and intense research [2].

The current prognostic factors are clinicopathological, notably based on the American Joint Committee on Cancer (AJCC) tumor, node and metastasis staging, and the criteria used for immediate surgery are technical (mainly based on the vascular involvement assessment), clinical (e.g., based on performance status), and biological (e.g., based on CA19-9 value). However, the criteria’s ability to consistently predict a patient’s outcome is limited, with substantial heterogeneity within the so-defined prognostic classes [11]. Actually, no prognostic or predictive biomarker has yet been established for pancreatic cancer. High-throughput molecular analyses revealed the extensive heterogeneity of cancers, and notably pancreatic cancer. Key molecular alterations have been identified, such as *KRAS*, *TP53*, *SMAD4*, *CDKN2A*, and *ARID1A* mutations and *GATA6* amplification [12, 13], but they remain without clinical application to date. Several studies of gene expression profiling have also been reported [14], mainly focused on the comparison of cancer versus normal pancreatic tissues. A few prognostic gene expression signatures have been developed [15–24], in general from small sample series and without validation in independent sets, or with validation in limited tumor sets. Biologically relevant molecular subtypes have been identified [16, 25, 26], and associated with OS [27]. However, identifying molecular predictors to aid in patient care remains necessary.

Here, we collected data of 695 pancreatic carcinoma samples from gene expression datasets, and searched for a gene expression signature predictive for post-operative OS.

## Methods

### Gene expression datasets

We retrospectively collected clinicopathological and gene expression data of clinical pancreatic carcinoma samples from nine publicly available datasets [15, 16, 20, 21, 23, 25, 28–30] from the National Center for Biotechnology Information/Genbank Gene Expression Omnibus, ArrayExpress, European Genome-phenome Archive, and The Cancer Genome Atlas (TCGA) databases (Additional file 1: Table S1). Samples had been profiled using whole-genome DNA microarrays (Affymetrix or Agilent) and RNA-Seq (Illumina). The complete dataset contained 695 samples, including 601 operated primary cancer samples with available survival data. The study was approved by our institutional board.

### Gene expression data analysis

Data analysis required pre-analytic processing. First, we normalized each DNA microarray-based dataset separately, by using quantile normalization for the available processed Agilent data, and Robust Multichip Average [31] with the non-parametric quantile algorithm for the raw Affymetrix data. Normalization was performed in R using Bioconductor and associated packages. Then, we mapped hybridization probes across the different technological platforms. We used SOURCE [32] and NCBI EntrezGene [33] to retrieve and update the Agilent annotations, and NetAffx Annotation files [34] for the Affymetrix annotations. The probes were then mapped according to their EntrezGeneID. When multiple probes represented the same GeneID, we retained the one with the highest variance in a particular dataset. For the TCGA, Bailey’s and Kirby’s data, we used the available normalized RNA-Seq data that we log_2_-transformed.

We defined the molecular subtypes of all pancreatic cancer samples in each dataset separately as defined in the original publications, i.e., the three Collisson’s subtypes [16] were classical, quasi-mesenchymal, and exocrine-like, the two Moffitt’s epithelial subtypes [26] were basal-like and classical, and the four Bailey’s subtypes [25] were squamous, pancreatic progenitor, immunogenic, and aberrantly differentiated endocrine exocrine (ADEX). To identify a prognostic expression signature, we applied a supervised analysis using learning and validation sets. The learning set was a subset (n = 39) of the Bailey’s and TCGA RNA-Seq datasets that included samples from patients with survival of at least 36 months after surgery (long-term survivors (LTS); n = 17) and from patients dead of disease between 2 and 6 months after surgery (short-term survivors (STS); n = 22). The 562 other samples with available survival data from the other datasets were gathered and used as an independent validation set. Samples of the learning set were pooled before supervised analysis by using COMBAT (empirical Bayes), included in the inSilicoMerging R/Bioconductor package, as a batch effects removal method. The final merged set included 15,291 genes in log2-transformed data. The accuracy of normalization was controlled by principal component analysis (Additional file 2: Figure S1). The supervised analysis compared the expression profiles of 15,291 genes between the 22 STS samples and the 17 LTS samples using a moderated *t*-test with empirical Bayes statistic included in the Limma R packages. False discovery rate was applied to correct for the multiple testing hypothesis and significant genes were defined by the following thresholds: *P* < 5%, false discovery rate < 25%, and fold change superior to |2x|. Ontology analysis of the resulting 1400-gene list was based on the gene ontology (GO) biological processes of the Database for Annotation, Visualization and Integrated Discovery (DAVID) [35]. We then developed a prognostic classifier while minimizing the number of retained genes. Starting from the resulting 1400-gene list, we used logistic regression analysis with Least Absolute Shrinkage and Selection Operator [36] (LASSO), which is a selection method that handles high-dimensional regression variables with no prior feature selection step by shrinking all regression coefficients toward zero, and thus forcing many regression variables to be exactly zero. The penalty regularization parameter λ was chosen via the cross-validation routine *cv.glmnet* before running the main algorithm implemented in the R package *glmnet* version 1.9-8, with an n-fold equal to 10. The λ value was finalized by using the lambda.min, which is the value of lambda giving minimum mean cross-validated error (lambda.min was 0.0153). The resulting classifier allowed the definition of two classes of samples, namely the predicted STS-like class and the predicted LTS-like class. Its robustness was assessed in the independent validation set (n = 562) by classifying each sample in each dataset separately as STS-like or LTS-like. Since a few studies have indicated that many gene signatures were random noise signatures [37, 38], we evaluated whether our prognostic 25-gene signature was not inferior to random signatures. A resampling scheme was used to generate 100,000 random 25-gene signatures within the 1400 genes differentially expressed identified by supervised analysis in the learning set. Each random signature was then applied to the validation set to determine its significance level in prognostic terms for OS. We then measured the proportion of random signatures with a *P* value inferior to the *P* value from our 25-gene signature.

### Statistical analysis

Associations between tumor groups and clinicopathological features were analyzed using the *t*-test or the Fisher’s exact test when appropriate. Overall survival (OS) was calculated from the date of diagnosis to the date of death from pancreatic cancer. Follow-up was measured from the date of diagnosis to the date of last news for living patients. Survivals were calculated using the Kaplan–Meier method and were compared with the log-rank test. Uni- and multivariate survival analyses were performed using Cox regression analysis (Wald test). Variables tested in univariate analyses included patient age at time of diagnosis (>60 vs. ≤ 60 years), sex (male vs. female), AJCC clinical stage (2, 3, and 4 vs. 1), pathological features including pathological type (others vs. ductal), tumor size (pT2, T3, and pT4 vs. T1), lymph node status (positive vs. negative), grade (2, 3, and 4 vs. 1), our 25-gene classification (STS-like vs. LTS-like), and the different molecular subtype classifications. Variables with a *P* value lower than 0.05 were tested in multivariate analysis. All statistical tests were two-sided at the 5% level of significance. Statistical analysis was performed using the survival package (version 2.30) in the R software (version 2.15.2) [39]. We followed the reporting REcommendations for tumor MARKer prognostic studies (REMARK criteria) [40]. A Sweave report describing the analysis of gene expression data and the associated statistical analysis is available as Additional file 3 (Supplementary Text).

## Results

### Patient population

We collected nine retrospective/prospective public whole-genome mRNA expression datasets of 695 pancreatic samples, and focused our analysis on the 601 cancer samples from patients operated from the outset and with available survival. As shown in Table 1, the majority of patients were aged 60 years or older, and 54% were male. Most cases (96%) were AJCC stage 1 or 2, ductal type (98%), and grade 2 (55%). All but one case had been treated by front-line surgery, and the majority of tumors were pT2 (16%) or pT3 (77%), and pN-positive (69%). All Bailey’s, Moffitt’s, and Collison’s molecular subtypes were represented. A total of 354 patients died. The median OS was 20 months (range, 1–156), and the 2-year OS was 40% (95% CI 36–45).

**Table 1:** Patient and tumor clinicopathological characteristics of 601 samples

Characteristics	All (*n* = 601)
Age at diagnosis, years	
≤ 60	118 (32%)
> 60	246 (68%)
Sex	
Female	170 (46%)
Male	197 (54%)
AJCC Stage	
1	62 (12%)
2	431 (84%)
3	10 (2%)
4	12 (2%)
Pathological type	
Ductal	537 (98%)
Other^a^	11 (2%)
Pathological grade	
1	33 (12%)
2	154 (55%)
3	91 (32%)
4	2 (1%)
Pathological tumor size (pT)	
pT1	18 (5%)
pT2	62 (16%)
pT3	302 (77%)
pT4	11 (3%)
Pathological lymph node status (pN)	
Negative	141 (31%)
Positive	310 (69%)
Collisson subtypes	
Classical	234 (39%)
Exocrine-like	211 (35%)
Quasi-mesenchymal	156 (26%)
Moffitt subtypes, ‘type’	
Basal-like	232 (39%)
Classical	369 (61%)
Bailey subtypes	
ADEX	140 (23%)
Immunogenic	104 (17%)
Pancreatic progenitor	142 (24%)
Squamous	215 (36%)
Deceased	354 (59%)
2-year OS (95% CI)	40% (36–45)
Median OS, months (range)	20 (1–156.4)

^a^Other: 8 neuroendocrine tumors, 2 acinar cell carcinomas, 1 intraductal tubulopapillary neoplasm

*ADEX* aberrantly differentiated endocrine exocrine, *AJCC* American Joint Committee on Cancer, *CI* confidence interval, *OS* overall survival

### Identification of a prognostic expression signature

We searched for a gene signature associated with OS. Supervised analysis was performed in a learning set of 39 samples selected to represent the two opposite groups of patients, including 17 LTS and 22 STS. Analysis identified 1400 genes differentially expressed between the two groups (Additional file 4: Table S2). All associated GO biological processes are shown in Additional file 5: Table S3, and the top 40 processes are shown in Table 2. The robustness of those genes was tested by testing their ability to classify the LTS and STS samples from the other independent datasets. Out of the 67 samples classified, 49 (76%) were accurately classified, suggesting strong robustness (*P* = 7.68 × 10^–5^, Fisher’s exact test).

**Table 2:** Top 40 gene ontology (GO) biological processes associated with the 1400 genes differentially expressed between the short-term survivor (STS) and long-term survivor (LTS) samples of the learning set

GO:BP TermsID	GO:BP Terms	*N*	*P* value	Status STS vs. LTS
GO:0030198	Extracellular matrix organization	70	9.15 × 10^–26^	Up
GO:0007155	Cell adhesion	75	2.46 × 10^–22^	Up
GO:0022617	Extracellular matrix disassembly	35	2.70 × 10^–18^	Up
GO:0008544	Epidermis development	25	1.04 × 10^–17^	Up
GO:0030574	Collagen catabolic process	26	2.78 × 10^–17^	Up
GO:0006955	Immune response	50	1.44 × 10^–16^	Up
GO:0006954	Inflammatory response	52	2.34 × 10^–16^	Up
GO:0030199	Collagen fibril organization	19	6.76 × 10^–16^	Up
GO:0018149	Peptide cross-linking	10	1.23 × 10^–10^	Up
GO:0006935	Chemotaxis	22	2.34 × 10^–10^	Up
GO:0010951	Negative regulation of endopeptidase activity	23	6.65 × 10^–10^	Up
GO:0030216	Keratinocyte differentiation	14	3.24 × 10^–9^	Up
GO:0010466	Negative regulation of peptidase activity	8	1.47 × 10^–8^	Up
GO:0001501	Skeletal system development	22	2.51 × 10^–8^	Up
GO:0007160	Cell-matrix adhesion	19	1.30 × 10^–7^	Up
GO:0000278	Mitotic cell cycle	50	1.71 × 10^–7^	Up
GO:0008283	Cell proliferation	42	1.86 × 10^–7^	Up
GO:0031124	Mrna 3-end processing	600	2.19 × 10^–7^	Up
GO:0008284	Positive regulation of cell proliferation	45	3.26 × 10^–7^	Up
GO:0001525	Angiogenesis	32	3.31 × 10^–7^	Up
GO:0019228	Neuronal action potential	6	1.26 × 10^–6^	Down
GO:0007409	Axonogenesis	12	1.13 × 10^–6^	Down
GO:0007628	Adult walking behavior	7	1.09 × 10^–6^	Down
GO:0007212	Dopamine receptor signaling pathway	5	7.83 × 10^–7^	Down
GO:0006906	Vesicle fusion	10	4.63 × 10^–7^	Down
GO:0030073	Insulin secretion	9	5.27 × 10^–8^	Down
GO:0007274	Neuromuscular synaptic transmission	6	5.20 × 10^–8^	Down
GO:0007399	Nervous system development	27	1.44 × 10^–8^	Down
GO:0014047	Glutamate secretion	9	5.89 × 10^–9^	Down
GO:0007626	Locomotory behavior	15	4.46 × 10^–9^	Down
GO:0086010	Membrane depolarization during action potential	8	1.71 × 10^–9^	Down
GO:0031018	Endocrine pancreas development	12	1.16 × 10^–9^	Down
GO:0017158	Regulation of calcium ion-dependent exocytosis	10	9.94 × 10^–10^	Down
GO:0006112	Energy reserve metabolic process	20	4.05 × 10^–11^	Down
GO:0017157	Regulation of exocytosis	13	1.60 × 10^–11^	Down
GO:0006813	Potassium ion transport	15	3.94 × 10^–12^	Down
GO:0071805	Potassium ion transmembrane transport	17	1.97 × 10^–13^	Down
GO:0016079	Synaptic vesicle exocytosis	17	4.00 × 10^–15^	Down
GO:0007269	Neurotransmitter secretion	19	7.04 × 10^–16^	Down
GO:0007268	Synaptic transmission	66	2.33 × 10^–37^	Down

To render this signature more easily applicable in clinics, we built a multigene classifier from the 1400-gene list. Logistic regression analysis retained 25 genes (Table 3), including 12 and 13 genes respectively upregulated and downregulated in the STS samples. As expected, the classifier based on these 25 genes sorted with 100% accuracy those 39 patients into two classes, with STS-like including all STS patients and LTS-like including all LTS patients.

**Table 3:** List of 25 genes included in our prognostic classifier

Symbol	Description	Cytoband	Expression status
GPR87	G protein-coupled receptor 87	3q24	Up STS vs. LTS
KRT13	keratin 13, type I	17q21.2	Up STS vs. LTS
RAC2	ras-related C3 botulinum toxin substrate 2 (rho family, small GTP binding protein Rac2)	22q13.1	Up STS vs. LTS
C16orf74	chromosome 16 open reading frame 74	16q24.1	Up STS vs. LTS
NAMPT	nicotinamide phosphoribosyltransferase	7q22.3	Up STS vs. LTS
DHRS9	dehydrogenase/reductase (SDR family) member 9	2q31.1	Up STS vs. LTS
HIST2H2BF	histone cluster 2, H2bf	1q21.2	Up STS vs. LTS
TREM2	triggering receptor expressed on myeloid cells 2	6p21.1	Up STS vs. LTS
ZDHHC20	zinc finger, DHHC-type containing 20	13q12.11	Up STS vs. LTS
CD180	CD180 molecule	5q12	Up STS vs. LTS
ADGRG6	adhesion G protein-coupled receptor G6	6q24.1	Up STS vs. LTS
APBB1IP	amyloid beta (A4) precursor protein-binding, family B, member 1 interacting protein	10p12.1	Up STS vs. LTS
EGR3	early growth response 3	8p23-p21	Down STS vs. LTS
MACROD2	MACRO domain containing 2	20p12.1	Down STS vs. LTS
EPHA7	EPH receptor A7	6q16.1	Down STS vs. LTS
RASGEF1A	RasGEF domain family, member 1A	10q11.21	Down STS vs. LTS
SYNM	synemin, intermediate filament protein	15q26.3	Down STS vs. LTS
S100A1	S100 calcium binding protein A1	1q21	Down STS vs. LTS
WNK2	WNK lysine deficient protein kinase 2	9q22.3	Down STS vs. LTS
RAMP2	receptor (G protein-coupled) activity modifying protein 2	17q12-q21.1	Down STS vs. LTS
SOCS2	suppressor of cytokine signaling 2	12q	Down STS vs. LTS
COL28A1	collagen, type XXVIII, alpha 1	7p21.3	Down STS vs. LTS
B4GALT6	UDP-Gal:betaGlcNAc beta 1,4-galactosyltransferase, polypeptide 6	18q11	Down STS vs. LTS
PLCB4	phospholipase C, beta 4	20p12	Down STS vs. LTS
MTURN	maturin, neural progenitor differentiation regulator homolog (Xenopus)	7p14.3	Down STS vs. LTS

We assessed the gene overlap between our 25-gene signature and the three molecular subtype classifiers [16, 25, 26] and five other signatures recently published that displayed robust and independent prognostic value [15, 17, 20, 22, 30]. As shown in Additional file 6: Figure S2, there was no overlap with the five signatures, and the overlap with the molecular subtype classifiers was very low (0 gene with Collisson, 1 with Moffitt stroma, 2 with Moffitt tumor, and 3 with Bailey).

### Validation of the 25-gene classifier and clinicopathological associations

**Fig. 1:**
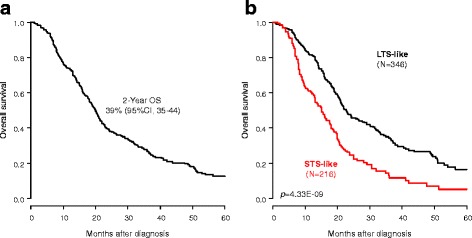
Overall survival (OS) in the validation set according to our prognostic 25-gene classifier. Kaplan–Meier OS curves in all patients (**a**) and in all patients according to our prognostic classifier (STS-like and LTS-like classes) (**b**). The *P* values of the log-rank test are indicated

We tested the 25-gene prognostic classifier in the independent validation set of 562 patients whose clinicopathological characteristics were close to those of the learning set (Additional file 7: Table S4) and with a 2-year OS of 39% (95% CI 35–44; Fig. 1a). The classifier sorted the 562 patients into two classes, STS-like (*n* = 216; 38%) and LTS-like (*n* = 346; 62%), with a 2-year OS of 25% (95% CI 18–33) and 48% (95% CI 42–54), respectively (*P* = 4.33 × 10^–9^, log-rank test; Fig. 1b), thus confirming its prognostic value. The respective median OS were 15 months (range, 1–104) and 23 months (range, 1–156). Interestingly, in each of the nine datasets separately, the 2-year OS was shorter in the STS-like class than in the LTS-like class, and the difference was or tended to be significant (Additional file 8: Figure S3). To assess the likelihood of our 25-gene signature as a non-random signature, we generated by a resampling scheme 100,000 random gene signatures from the list of 1400 genes differentially expressed and tested their prognostic value in the validation set. None of the random signatures was more significant than the data-derived 25-gene signature, suggesting that the latter represented an optimal prognostic combination.

We searched for associations between the 25-gene signature-based classification and the clinicopathological variables of samples. As shown in Table 4, no association was found with patient age and sex, AJCC stage, pathological type, tumor size, and lymph node status. By contrast, there were more grade 3 tumors (*P* = 1.50 × 10^–3^) in the STS-like class, and more aggressive molecular subtypes (Baileys’ squamous, Collison’ quasi-mesenchymal, and Moffitt’s basal-like; *P* < 0.05).

**Table 4:** Associations of our prognostic classification with clinicopathological features (validation set)

Characteristics	*n*	LTS-like (*n* = 346)	STS-like (*n* = 216)	*P* value
Age at diagnosis				0.219
≤ 60	108	64 (31%)	44 (38%)	
> 60	217	145 (69%)	72 (62%)	
Sex				0.419
Female	157	105 (50%)	52 (44%)	
Male	171	106 (50%)	65 (56%)	
AJCC stage				0.759
1	54	35 (12%)	19 (11%)	
2	403	255 (84%)	148 (85%)	
3	10	5 (2%)	5 (3%)	
4	11	8 (3%)	3 (2%)	
Pathological type				0.087
Ductal	504	308 (98%)	196 (100%)	
Other	6	6 (2%)	0 (0%)	
Pathological grade				1.50 × 10^–3*^
1	27	24 (16%)	3 (3%)	
2	138	90 (59%)	48 (54%)	
3	75	38 (25%)	37 (42%)	
4	2	1 (1%)	1 (1%)	
Pathological tumor size (pT)				0.879
pT1	15	9 (4%)	6 (5%)	
pT2	57	36 (16%)	21 (16%)	
pT3	281	181 (78%)	100 (76%)	
pT4	11	6 (3%)	5 (4%)	
Pathological lymph node status (pN)				0.824
Negative	123	79 (30%)	44 (29%)	
Positive	291	183 (70%)	108 (71%)	
Collisson subtypes				1.00 × 10^–6*^
Classical	223	130 (38%)	93 (43%)	
Exocrine-like	194	147 (42%)	47 (22%)	
Quasi-mesenchymal	145	69 (20%)	76 (35%)	
Moffitt subtypes, ‘type’				7.80 × 10^–15*^
Basal-like	214	88 (25%)	126 (58%)	
Classical	348	258 (75%)	90 (42%)	
Bailey subtypes				1.00 × 10^–6*^
ADEX	128	114 (33%)	14 (6%)	
Immunogenic	101	69 (20%)	32 (15%)	
Pancreatic progenitor	133	90 (26%)	43 (20%)	
Squamous	200	73 (21%)	127 (59%)	
2-year OS (95% CI)	562	48% (42–54)	25% (0.18–0.33)	4.33 × 10^–9*^
Median OS, months (range)	562	22.8 (1–156.4)	15.0 (1–103.92)	

*ADEX* aberrantly differentiated endocrine exocrine, *CI* confidence interval, *LTS* long-term survivors, *STS* short-term survivors, *OS* overall survival

*Statistically significant

### Uni- and multivariate prognostic analyses

We compared the prognostic value of our 25-gene classifier with that of other clinicopathological variables in the validation set. In univariate analysis (Table 5), three variables were associated with OS (Wald test), namely the AJCC clinical stage (*P* = 4.71 × 10^–3^), the pathological pN status (*P* = 1.24 × 10^–4^), and our 25-gene classifier (*P* = 7.47 × 10^–9^). The hazard ratio (HR) for death was 1.93 (95% CI 1.55–2.42) in the STS-like vs. LTS-like classes. In multivariate analysis, only our classifier (*P* = 6.33 × 10^–7^) and the pN status (*P* = 2.95 × 10^–2^) remained significant, suggesting an independent prognostic value. The stratification of patients according to both the classifier and the AJCC stage identified classes with different 2-year OS (Additional file 9: Figure S4). For example, in patients with stage 1 tumor, the 2-year OS was 42% in the STS-like class (42%) and 73% in the LTS-like class (*P* = 6.74 × 10^–3^, log-rank test). Stage 2 patients were similarly subdivided into STS- and LTS-like with a 21% and 46% 2-year OS (*P* = 4.37 × 10^–7^, log-rank test), respectively.

**Table 5:** Uni- and multivariate Cox regression analyses for overall survival (validation set)

Characteristics		Univariate			Multivariate			Multivariate		
		*N*	HR (95% CI)	*P* value	*N*	HR (95% CI)	*P* value	*N*	HR (95% CI)	*P* value
Age at diagnosis	>60 vs. ≤ 60	325	1.22 (0.88–1.70)	0.234						
Sex	Male vs. female	328	1.08 (0.80–1.45)	0.633						
AJCC Stage	2 vs. 1	478	2.01 (1.32–3.07)	4.71 × 10^–3*^	408	1.57 (0.88–2.82)	0.128			
	3 vs. 1		3.11 (1.33–7.23)		408	2.21 (0.82–5.97)	0.119			
	4 vs. 1		2.85 (1.16–7.05)		408	1.44 (0.19–10.97)	0.723			
Pathological type	Other vs. ductal	510	0.36 (0.09–1.45)	0.151						
Pathological grade	2 vs. 1	242	1.52 (0.65– 3.55)	0.185						
	3 vs. 1		2.15 (0.91– 5.11)							
	4 vs. 1		2.66 (0.53–13.3)							
Pathological tumor size (pT)	2 vs. 1	364	1.49 (0.62–3.59)	0.131						
	3 vs. 1		1.95 (0.86–4.42)							
	4 vs. 1		2.93 (1.01–8.48)							
Pathological lymph node status (pN)	1 vs. 0	414	1.83 (1.34–2.48)	1.24 × 10^–4*^	408	1.50 (1.04–2.16)	2.95 × 10^–2*^			
Collisson subtypes	Exocrine-like vs. classical	562	1.00 (0.77–1.29)	2.32 × 10^–3*^				562	0.94 (0.66–1.34)	0.732
	Quasi-mesenchymal vs. classical		1.52 (1.17–1.99)					562	1.15 (0.83–1.59)	0.395
Moffitt subtypes, ‘type’	Classical vs. basal-like	562	0.64 (0.51–0.80)	6.29 × 10^–5*^				562	1.00 (0.72–1.38)	0.994
Bailey subtypes	Immunogenic vs. ADEX	562	0.81 (0.57–1.17)	8.98 × 10^–6*^				562	0.68 (0.43–1.06)	0.090
	Pancreatic progenitor vs. ADEX		0.97 (0.70–1.35)					562	0.79 (0.51–1.23)	0.302
	Squamous vs. ADEX		1.64 (1.22–2.19)					562	1.09 (0.68–1.74)	0.731
25-gene classifier	STS-like vs. LTS-like	562	1.93 (1.55–2.42)	7.47 × 10^–9*^	408	2.04 (1.54–2.70)	6.33 × 10^–7*^	562	1.77 (1.38–2.26)	6.33 × 10^–6*^

*ADEX* aberrantly differentiated endocrine exocrine, *CI* confidence interval, *HR* hazard ration, *LTS* long-term survivors, *STS* short-term survivors

*Statistically significant

**Fig. 2:**
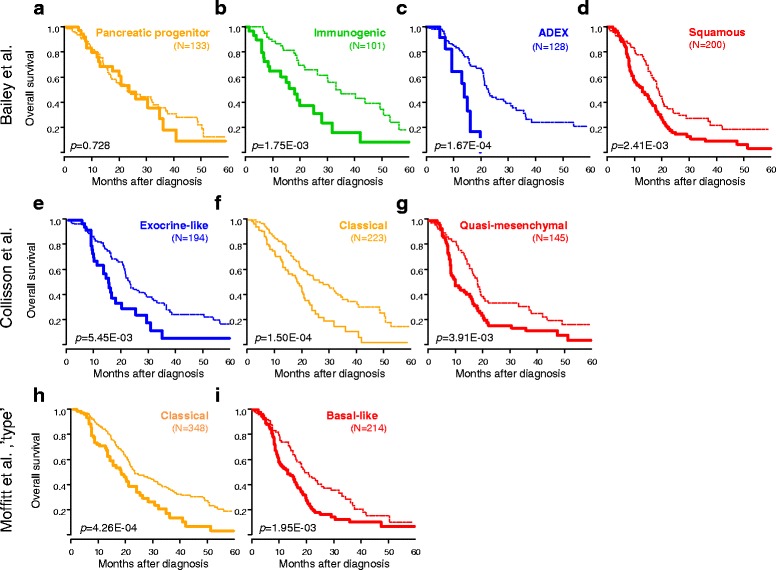
Overall survival (OS) in the validation set according to our prognostic 25-gene classifier and the molecular subtypes. Kaplan–Meier OS curves according to our prognostic classifier (STS-like and LTS-like classes) and the molecular subtypes defined by Bailey (**a** pancreatic progenitor; **b** immunogenic; **c** ADEX; **d** squamous), Collison (**e** exocrine-like; **f** classical; **g** quasi-mesenchymal), and Moffitt (**h** classical; **i** basal-like). The *P* values (log-rank test) for the comparison between the two classes within each molecular subtype are indicated

Given the association between the molecular subtypes and the 25-gene classifier, we compared their respective prognostic performance. In univariate analysis, the three molecular subtype classifiers confirmed their prognostic value in this large sample set (Additional file 10: Figure S5). However, in multivariate analysis including the four multigene classifiers, only our 25-gene classifier remained significant (*P* = 6.33 × 10^–6^, Wald test, Table 5) with a HR of 1.77 (95% CI 1.38–2.26). As shown in Fig. 2, it affected the clinical outcome of all molecular subtypes of all three classifications, except the Bailey’s progenitor subtype.

## Discussion

Pancreatic carcinoma is a heterogeneous disease with high metastatic propensity and poor prognosis. In patients with resectable disease, the development of effective systemic therapies is crucial. During the last decades, several retrospective studies [41] and a few prospective phase II studies [8–10] have suggested the potential benefit of neoadjuvant chemotherapy, and large randomized phase II/III trials are ongoing. In this context, a major challenge is to improve the imperfect current prognostic factors to aid in therapeutic decision-making, notably regarding the decision for immediate surgery followed by chemotherapy or neoadjuvant chemotherapy followed by surgery. Here, we have analyzed whole-genome expression profiles of 601 pancreatic carcinoma samples from operated patients, and identified a robust 25-gene classifier associated with post-operative OS independently of classical prognostic factors and molecular subtypes. To our knowledge, this study is by far the largest prognostic study of gene expression profiles in pancreatic carcinoma.

Gene expression profiling remains today the most promising and successful high-throughput molecular approach to identify new prognostic tools in early-stage cancers. Multigene signatures are already marketed, such as Oncotype™ in breast cancer or Coloprint™ in colon cancer, yet no similar signature is available in pancreatic carcinoma. The paucity of tumor specimens available for analysis explains the relatively small number of samples profiled in previous prognostic studies, with 102 samples in the largest one [20] to use supervised analysis, and 328 in the Australian ICGC study [25], which identified prognostic molecular subtypes by unsupervised analysis. We overcame the problem by pooling nine public datasets, representing a total of 601 operated primary cancers with available follow-up, and allowing the use of a learning set and a validation set in the supervised analysis. Our series displayed classical clinicopathological characteristics and poor prognosis with a 40% 2-year OS. The learning set, which included only 39 samples, was remarkably small compared with the validation set; this might have reduced our ability to capture the best genes for the classifier. However, it was carefully designed to contain two groups with distinct aggressiveness, namely a LTS group after surgery and a STS group, and to contain samples profiled using the same technology (RNA-Seq). Such design likely explains the large number of genes (1400) differentially expressed between the two patient groups despite the correction for the multiple testing hypothesis, and the robustness of our final signature in the validation set. A similar design had been used previously [20] by comparing primary tumors from metastatic versus non-metastatic patients. The size of our series allowed testing of the classifier in a large independent validation set of 562 samples with multivariate analysis and increased statistical power. For comparison, the other prognostic expression signatures published to date in pancreatic cancer [15–24] were defined in learning sets including 6–70 clinical samples, then tested in validation sets including 67–246 samples, with inconstant multivariate analysis.

We first identified 1400 genes differentially expressed between the STS and LTS samples. From this gene list, a 25-gene classifier was developed, identifying two classes, namely STS-like and LTS-like. The prognostic value was verified in the independent validation set, in which the two classes, STS-like (38% of samples) and LTS-like (62%), showed a different 2-year OS (25% in the STS-like and 48% in the LTS-like). Interestingly, and by contrast to the other published studies in the field, this prognostic value existed in each of the nine datasets considered separately. As expected, the other variables significant in univariate analysis included the AJCC stage and the pathological lymph node status. The pathological type (other vs. ductal) was not significant (HR 0.36 with *P* = 0.151) because of the small percentage (1%) of “other” types. Interestingly, all six “other” type samples were classified in the LTS-like class, in agreement with the better prognosis of neuroendocrine tumors. Importantly, the OS analysis was not modified when limited to the 504 ductal samples with a 27% 2-year OS in the STS-like and 48% in the LTS-like. Our 25-gene classifier displayed an independent prognostic value. Interestingly, it outperformed the molecular subtypes in multivariate analysis and identified patients with shorter and longer survival in all subtypes but one, highlighting substantial heterogeneity in each of them. None of the 100,000 25-gene signatures randomly generated by a resampling scheme was more significant than the data-derived 25-gene signature, suggesting that this latter represented a non-random optimal prognostic combination.

Ontology analysis of the 25 genes revealed interesting pathways, such as pathways related to the metastatic process (extracellular matrix organization and disassembly, cell and cell-matrix adhesion), local inflammation (immune and inflammatory responses, chemotaxis), and cell proliferation (mitotic cell cycle, positive regulation of proliferation) associated with the “poor-prognosis genes”. Pathways associated with the “good-prognosis genes” included those related to pancreas metabolism (endocrine pancreas development, energy reserve metabolic process, insulin secretion) or synaptic connections (synaptic transmission and vesicle exocytosis, membrane depolarization during action potential). Whether the 25 classifier genes are causative of the phenotype in a biological sense or reflect another associated phenomenon remain to be explored. However, it was interesting to find some genes already reported as associated with cancer biology and/or to the clinical outcome of cancer patients. Among the genes upregulated in STS, *GPR87*, *RAC2*, *NAMPT*, *C16orf74*, *TREM2*, and *CD180* are involved in NF-_K_B-mediated cell signaling, and *KRT13*, *RAC2*, *C16orf74*, *ADGRG6*, and *APBB1IP* in epithelial–mesenchymal transition. These two pathways are frequently affected in pancreatic ductal adenocarcinoma (PDAC) [42, 43]. Activation of the NF-_K_B signaling pathway plays an important role in the development and progression of disease and impacts the epithelial–mesenchymal transition, chemoresistance, migration, and invasion of pancreatic cancer cells [42, 44–46]. The NF-_K_B activation pathway picked by our signature might not necessarily be related to tumor cells themselves. Stromal cells can modulate their activation status through NF-_K_B, based on the signals collected from their environment. TREM2 and CD180 are negative regulators of the Toll-like receptor pathway [47], a family of receptors that recognize damage-associated molecule patterns, whose increased serum levels have been associated with cancer [48]. Inhibition of Toll-like receptors results in impaired immediate host defensive responses and anti-tumor response mounting. TREM2 and CD180 are also part of the conventional markers used to describe “alternatively” activated M2 macrophages. M2 macrophages promote angiogenesis, tissue remodeling and repair, thus facilitating tumor progression and invasion, and their presence is correlated with poor prognosis in several cancers, including PDAC [49, 50]. Identifying molecules that modulate some specific “activation nodes” of the wide NF-_K_B signaling pathway could be interesting for pancreatic cancer therapy. Two other genes related to NF-_K_B activation are *GPR87* and *NAMPT*, and represent potential therapeutic targets. *GPR87* is overexpressed in various cancers, including pancreatic cancer cells and tissues, and its overexpression correlates with shorter OS [51]. GPR87 enhances pancreatic cancer aggressiveness by activating the NF-_K_B signaling pathway, and plays a role in tumor cell survival [52, 53] and the regulation of TP53 [54]. Antagonists of GPR87 are in development [53]. *NAMPT* is one of the two enzymes regulating the NAD+ salvage pathway, a vital pathway allowing pancreatic cancer cells to maintain their metabolism, notably in hypoxic conditions [55]. *NAMPT* is also involved in tumor angiogenesis [56, 57]. Thus, targeting *NAMPT* may not only disturb the salvage pathway on which pancreatic tumor cells heavily rely, but may also “normalize” blood vessels in the tumor, a phenomenon that will improve the delivery and efficacy of anticancer treatments and relieve immunosuppression [58, 59]. Several NAMPT inhibitors are currently in development in oncology [60]. For example, FK866, a non-competitive highly specific inhibitor of NAMPT, shows potent anti-tumor activity both in vitro and in vivo [61] on pancreatic cancer samples overexpressing *NAMPT* mRNA. Among the other genes of our signature upregulated in STS samples are *C16orf74* and *KRT13*, which are associated with poor OS in pancreatic [62] and prostate [63] cancers.

Thirteen genes of our signature were downregulated in STS samples. Three of them, *EGR3*, *EPHA7*, and *MACROD2*, play a role in peripheral nervous system biology, which may have a role in PDAC aggressiveness [64]. We previously reported that the *MACROD2* locus at chromosome 20p12.1 may be a cancer-specific fragile site often affected in PDAC [65]. Four genes (*EPHA7*, *SOCS2*, *SYNM*, *WNK2*) are tumor suppressor genes whose hypermethylation is a common mechanism of downregulation. WNK2 is a serine-threonine kinase involved in the regulation of electrolyte homeostasis, cell survival, and proliferation. Its downregulation occurs early in PDAC oncogenesis [66]. SOCS2 is an important regulator of the JAK-STAT pathway [67]. SYNM is a type IV intermediate filament involved in the modulation of cell adhesion and motility; in breast cancer, SYNM methylation is associated with shorter recurrence-free survival [68].

## Conclusions

We have identified a 25-gene classifier associated with post-operative OS independently of classical prognostic factors and molecular subtypes. The strength of our study lies in the size of the series, the robustness of the classifier in a large and multicentric validation set and in each dataset separately, its independent prognostic value, its non-random nature, and the biological relevance of the included genes. The small number of genes should facilitate the clinical application of the classifier by using other transcriptional tests applicable to formaldehyde-fixed paraffin-embedded samples such as qRT-PCR, RNAscope™ or Nanostring™ technologies. Limitations include the retrospective nature of our series and associated biases. Despite the very high *P* values, the HR for death was relatively low, around 2, in both uni- and multivariate analyses, and therefore of uncertain clinical value. However, we think that the testing of our signature in the current prospective trials of adjuvant and neoadjuvant chemotherapy trials is warranted, and should be tested not only as a two-tiered classifier, but also as a continuous score. Indeed, a continuous score based on the expression of 25 genes showed significant prognostic value (data not shown) in univariate analysis (HR for death of 2.84 (95% CI 2.06–3.91), *P* = 1.96 × 10^–10^) and in multivariate analysis (HR for death of 3.25 (95% CI 2.11–4.99), *P* = 7.42 × 10^–8^). If validated, our signature could help select patients with resectable disease for either immediate surgery (for the predicted LTS-like patients) or neoadjuvant chemotherapy (for the predicted STS-like patients), which ultimately should affect outcome and impact quality of life. Of course, the clinical utility of this approach will have to be prospectively demonstrated prior to any use in clinical routine. Neoadjuvant chemotherapy, currently mainly based on anatomical considerations, might also be indicated, and its benefits maximized, on the basis of the expression profile of aggressiveness, regardless of resectability. Finally, some of the classifier genes, or the pathways in which they are involved, may represent therapeutic targets. Therefore, functional studies to assess this are warranted.

## Additional files

Additional file 1: Table S1. List of pancreatic cancer datasets included in our analysis. List of pancreatic cancer datasets included in our analysis. (XLS 28 kb)

Additional file 2: Figure S1. Principal component analysis (PCA) of pancreatic carcinoma samples of the learning set before and after normalization. PCA was applied to the 279 TCGA and ICGC samples and the 685 Bailey’s classifier genes. Before normalization (A), samples are grouped in the 2D scatter plot representation according to their origin dataset (left), and not according to their Bailey’s molecular subtype type (right), whereas after normalization (B), all samples are grouped according to their molecular subtype (right), and not according to their origin dataset (left), suggesting that the inter-set technical differences have been removed by normalization. In A and B, each colour represents a set (left) and each colour represents a molecular subtype (right). (PPTX 595 kb)

Additional file 3: Supplementary Text. Sweave report. Sweave report describing the different steps of gene expression data analysis and associated statistics. (PDF 1013 kb)

Additional file 4: Table S2. List of 1400 genes differentially expressed between the short-term survivor (STS) samples and long-term survivor (LTS) samples of the learning set. List of 1400 genes differentially expressed between the STS and LTS samples of the learning set. (XLS 382 kb)

Additional file 5: Table S3. Ontology analysis of the 1400 genes differentially expressed between the short-term survivor (STS) and long-term survivor (LTS) samples of the learning set. Ontology analysis of the 1400 genes differentially expressed between the STS and LTS samples of the learning set. (XLS 3728 kb)

Additional file 6: Figure S2. Gene overlap between our 25-gene signature and other prognostic signatures. Venn diagram showing the overlap in genes between our signature and three prognostic signatures (A, Wang’s 28-gene signature, Haider’s 36-gene signature, and Chen’s 15-gene signature; the Stratford’s 6-gene and the Kirby’s 19-gene signatures are not shown because they display no gene common with the other four signatures), and between our signature and the four molecular subtype classifiers (B, Bailey’s 859-gene classifier, Collisson’s 62-gene classifier, Moffitt’s tumor 50-gene classifier, and Moffitt’s stroma 48-gene classifier). (PPTX 131 kb)

Additional file 7: Table S4. Patients and tumor clinicopathological characteristics of the learning and validation sets. (XLS 31 kb)

Additional file 8: Figure S3. Overall survival (OS) in each set of the pooled validation set according to our prognostic 25-gene classifier. Kaplan–Meier OS curves in all patients according to our prognostic classifier (STS-like and LTS-like classes). The dashed vertical line represents the 2-year OS. The *P* values of the log-rank test are indicated. (PPTX 142 kb)

Additional file 9: Figure S4. Overall survival (OS) in the validation set according to our prognostic 25-gene classifier and the American Joint Committee on Cancer (AJCC) Tumor, Node and Metastasis stage. Kaplan–Meier OS curves according to our prognostic classifier (STS-like and LTS-like classes) in patients with AJCC stage 1 (a) and AJCC stage 2 (b). The *P* values of the log-rank test are indicated. (PPTX 78 kb)

Additional file 10: Figure S5. Overall survival (OS) in the validation set according to the molecular subtypes. Kaplan–Meier OS curves according to the molecular subtypes defined by Bailey (a), Collison (b), and Moffitt (c). The *P* values of the log-rank test are indicated. (PPTX 103 kb)

## Abbreviations

ADEX: aberrantly differentiated endocrine exocrine
AJCC: American Joint Committee on Cancer
GO: gene ontology
HR: hazard ratio
LTS: long-term survivor
OS: overall survival
PDAC: pancreatic ductal adenocarcinoma
STS: short-term survivor
TCGA: The Cancer Genome Atlas
