# Supplementary material for: A 25-gene classifier predicts overall survival in resectable pancreatic cancer
Source: BMC Med. 2017 Sep 20;15:170. doi: 10.1186/s12916-017-0936-z (PMC5606023; doi:10.1186/s12916-017-0936-z)
Supplement: Supplementary file 2 — Principal component analysis (PCA) of pancreatic carcinoma samples of the learning set before and after normalization. PCA was applied to the 279 TCGA and ICGC samples and the 685 Bailey’s classifier genes. Before normalization (A), samples are grouped in the 2D scatter plot representation according to their origin dataset (left), and not according to their Bailey’s molecular subtype type (right), whereas after normalization (B), all samples are grouped according to their molecular subtype (right), and not according to their origin dataset (left), suggesting that the inter-set technical differences have been removed by normalization. In A and B, each colour represents a set (left) and each colour represents a molecular subtype (right). (PPTX 595 kb) [file 12916_2017_936_MOESM2_ESM.pptx]

## Slide 1
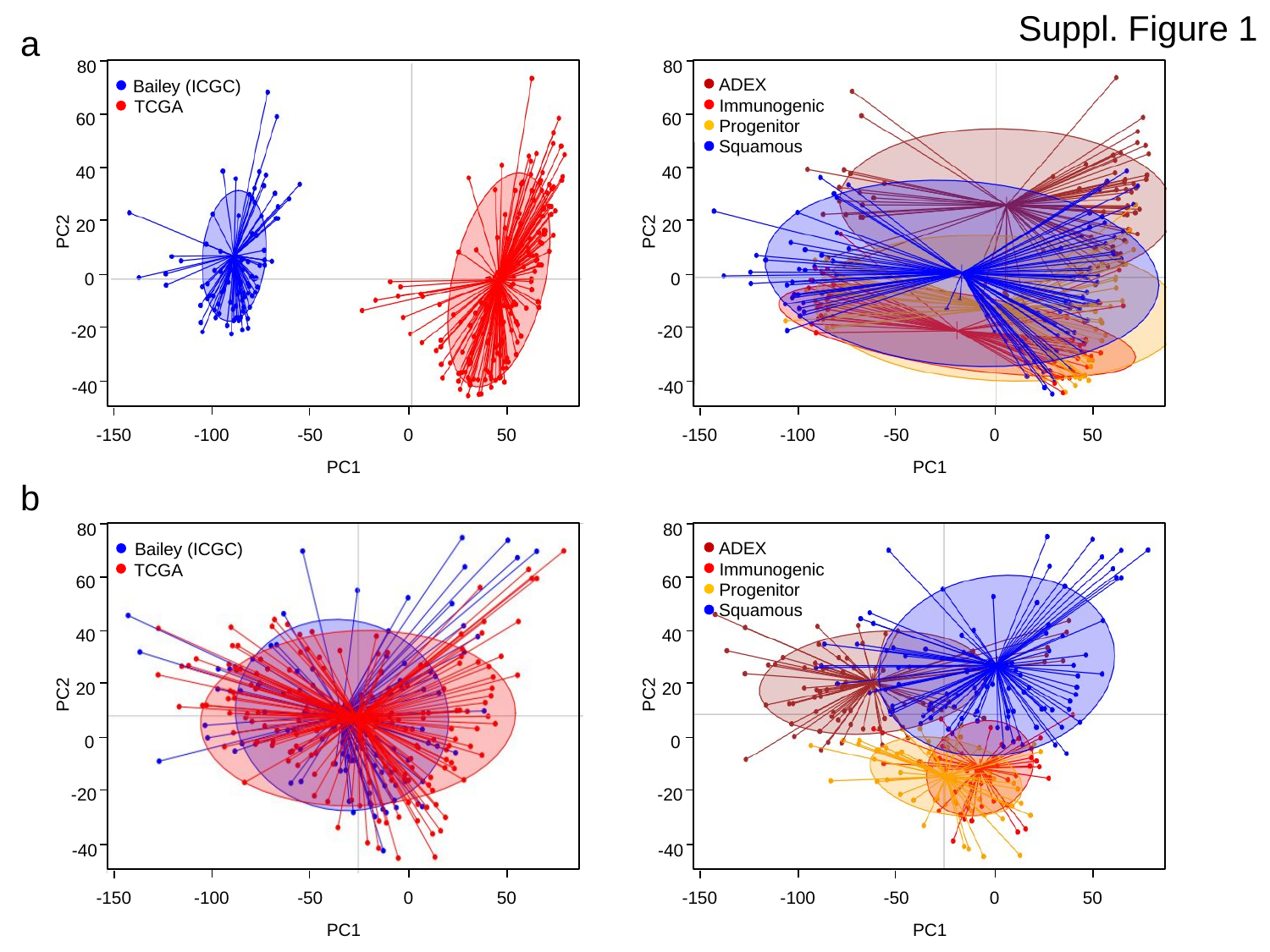

Suppl. Figure 1
a
80
60
40
20
PC2
0
-20
-40
-150
-100
-50
0
50
PC1
80
60
40
20
PC2
0
-20
-40
-150
-100
-50
0
50
PC1
ADEX
Immunogenic
Progenitor
Squamous
Bailey (ICGC)
TCGA
b
80
60
40
20
PC2
0
-20
-40
-150
-100
-50
0
50
PC1
80
60
40
20
PC2
0
-20
-40
-150
-100
-50
0
50
PC1
ADEX
Immunogenic
Progenitor
Squamous
Bailey (ICGC)
TCGA
