# Supplementary material for: A 25-gene classifier predicts overall survival in resectable pancreatic cancer
Source: BMC Med. 2017 Sep 20;15:170. doi: 10.1186/s12916-017-0936-z (PMC5606023; doi:10.1186/s12916-017-0936-z)
Supplement: Supplementary file 3 — Supplementary Text. Sweave report. Sweave report describing the different steps of gene expression data analysis and associated statistics. (PDF 1013 kb) [file 12916_2017_936_MOESM3_ESM.pdf]

A 25-gene classifier predicts overall survival in resectable pancreatic cancer

...

Sweave report

...

David J Birnbaum, Pascal Finetti, Alexia Lopresti,
Marina Gilabert, Flora Poizat, Jean-Luc Raoul,
Jean-Robert Delpero, Vincent Moutardier, Daniel Birnbaum,
Emilie Mamessier, François Bertucci

1 Preparation of public data

1.1 Loading description file sets with their location:

```
> library(xtable)
> Path <- "G:\\Pancreas"
> setwd(paste(Path, "GES_surv", sep="\\"))
> Set <- read.delim(paste(Path, "Description_SetExt.eSet_PA.txt", sep="\\"))
> print(xtable(Set[,c(1:4)]), size="footnotesize",include.rownames = FALSE)
```

Cohort	DataSet_ID	Platform	FileName
Stratford2010	GSE21501	Agilent-014850 WHG 4x44K	2010_Straford_GSE21501_S4.RData
vdBroeck2012	GSE42952	Affymetrix HG-U133 Plus 2.0	2012_vdBroeck_GSE42952_S4.RData
Zhang2012	GSE28735	Affymetrix HG-1.0 ST	2012_Zhang_GSE28735_S4.RData
Winter2012	E-MEXP-2780	Affymetrix HG-U133 Plus 2.0	2012_Winter_E_MEXP_2780_S4.RData
TCGA2015	TCGA_PAAD	RNAseq Illumina v3	20151020_PAAD_TCGA_RNAseqV2_S4.RData
Bailey2016	Bailey_ICGC-PACA-AU	RNAseq Illumina	2016_Bailey_Nat2016_S4.RData
Chen2015	GSE57495	Affymetrix, custom	2015_Chen_PAAD_GSE57495_RMA_S4.RData
Kirby2016	GSE79668	RNAseq, Illumina High-Seq	2016_Kirby_GSE79668_S4.RData
Collisson2013	GSE17891	Affymetrix HG-U133 Plus 2.0	2013_Collisson_GSE17891_RMAS4.RData

Public sets[1]

1.2 Merging phenodata from .RData objects

```
> PublicSet <- list(HC=c()) ; require(affy)
> for (i in seq_along(Set$Nom_ObjHC)){
+   load(paste(Path, "Rdata_ObjSets", Set$FileName[i], sep="\\"))
+   PublicSet$HC <- rbind(PublicSet$HC, pData(Eset_Obj)) ; rm(Eset_Obj)
+ }
> print(xtable(data.frame(table(PublicSet$HC$SetName)),
+              caption="Merged phenodata effectif"),
+       size="footnotesize",
+       include.rownames = FALSE)
```

Var1	Freq
2010_Straford_GSE21501_S4	132
2012_vdBroeck_GSE42952_S4	23
2012_Winter_E_MEXP_2780_S4	30
2012_Zhang_GSE28735_S4	90
2013_Collisson_GSE17891_RMAS4	27
2015_Chen_PAAD_GSE57495_RMA_S4	63
20151020_PAAD_TCGA_RNAseqV2_S4	183
2016_Bailey_Nat2016_S4	96
2016_Kirby_GSE79668_S4	51

Merged phenodata effectif

[1]Each of .RData objects listed were previously constructed from expression data and phenodata available. Phenodata were cleaned and homogenized across data sets. For Agilent-based data sets, we applied quantile normalization (*limma* package) to processed data. For Affymetrix-based data sets, we used Robust Multichip Average (RMA, *affy* package) on the raw .CEL files. Updated genechips annotation were integrated in their respective .RData object.

1.3 Selection of primary PACA samples w/ survival and w/o duplicate mesurement

```
> # Overall survival (OS)
> sel_Primaire <- which(PublicSet$HC$Type %in% "Primary" &
+                         PublicSet$HC$Duplicate %in% "unique" &
+                         PublicSet$HC$OS.Evt >= 0 & PublicSet$HC$OS.Del >= 0)
> length(sel_Primaire)
```

```
[1] 601
```

1.4 Variables preparation and groups selection

Variables list

```
> VAR_List <- as.list(PublicSet$HC)
> VAR_ListSurv <- list(OS=list(Evt=PublicSet$HC$OS.Evt, Del=PublicSet$HC$OS.Del))
```

Definition STS and LTS groups, i.e. event <= 6 months and censorship >= 36 months

```
> GRP_STS6m36m <- rep(NA, nrow(PublicSet$HC))
> GRP_STS6m36m[intersect(sel_Primaire,
+                      which(VAR_ListSurv$OS$Evt == 1 & VAR_ListSurv$OS$Del <= 6))] <- "STS"
> GRP_STS6m36m[intersect(sel_Primaire,
+                      which(VAR_ListSurv$OS$Evt == 0 & VAR_ListSurv$OS$Del >= 36))] <- "LTS"
```

Selection learning set : Bailey and TCGA sets, STS and LTS groups (N=39)

```
> selLearn <- intersect(sel_Primaire,
+                       which(PublicSet$HC$Cohort %in% c("Bailey2016", "TCGA2015") &
+                                  ((VAR_ListSurv$OS$Evt == 1 & VAR_ListSurv$OS$Del <= 6) |
+                                  (VAR_ListSurv$OS$Evt == 0 & VAR_ListSurv$OS$Del >= 36))))
```

Selection validation set : all remaining primary PACA (N=562)

```
> sel562 <- setdiff(sel_Primaire, selLearn)
```

Effectif learning and validation cohorts

```
> GRP_Learn_val <- rep(NA, nrow(PublicSet$HC))
> GRP_Learn_val[selLearn] <- "Learning"
> GRP_Learn_val[sel562] <- "Validation"
> print(xtable(data.frame(table(PublicSet$HC$Cohort[sel_Primaire],
+                     GRP_Learn_val[sel_Primaire])[c(1,6,2:5,7:9),]),
+               caption="Learning and validation cohort"),
+       size="footnotesize",
+       include.rownames = TRUE)
```

	Learning	Validation
Bailey2016	17	79
TCGA2015	22	156
Chen2015	0	63
Collisson2013	0	27
Kirby2016	0	51
Stratford2010	0	102
vdBroeck2012	0	12
Winter2012	0	30
Zhang2012	0	42

Learning and validation cohort

2 Molecular subtypes classification of public data

2.1 Bailey, Collisson and Moffitt classifications, 9 data sets

```
> RES_List <- list()
> for ( i in 1:nrow(Set)){
+   load(paste(Path, "RData_ObjSets", Set$FileName[i], sep="\\"))
+   #### import chip annot
+   Annotation <- annotation(Eset_Obj)
+   load(paste(Path, "RData_ObjSets\\chip", Annotation, ".RData", sep=""))
+   CHIP <- CHIP[match(featureNames(Eset_Obj), CHIP$PID),]  # internal ctrl
+   #### data matrix
+   DATA <- exprs(Eset_Obj)
+   #### Classification :
+   ### Baileys molecular subtypes
+   source("g:/Ori Tools/Prog/Rwork/Script/Classifier/20160607_Bailey_PAAD_4KssType.r")
+   Bailey_Tmp <- Bailey.PF(DATA, CHIP$GeneID, Set$Nom_ObjHC[i])
+   RES_Bailey_Tmp <- data.frame(Bailey_Tmp)
+   colnames(RES_Bailey_Tmp) <- paste("Bailey", colnames(RES_Bailey_Tmp), sep="_")
+   ### Collissons molecular subtypes
+   source("g:/Ori Tools/Prog/Rwork/Script/Classifier/20160930_Collisson_PAAD_3KssType.r")
+   Collisson_Tmp <- Collisson_PAAD.PF( DATA, CHIP$GeneID, Set$Nom_ObjHC[i])
+   RES_Collisson_Tmp <- data.frame(Collisson_Tmp)
+   colnames(RES_Collisson_Tmp) <- paste("Collisson", colnames(RES_Collisson_Tmp), sep="_")
+   ### Moffitts molecular subtypes
+   source("g:/Ori Tools/Prog/Rwork/Script/Classifier/20161003_Moffitt_PAAD_2x2KssType.r")
+   Moffitt_Tmp <- Moffitt_PAAD.PF(DATA, CHIP$GeneID, Set$Nom_ObjHC[i])
+   RES_Moffitt_Tmp <- data.frame(Moffitt_Tmp)
+   colnames(RES_Moffitt_Tmp) <- paste("Moffitt", colnames(RES_Moffitt_Tmp), sep="_")
+   ###
+   rm(Eset_Obj, DATA, CHIP)
+ }
> #### Merge classifications
> RES_Pool <- c()
> for (i in 1:length(RES_List)){
+     RES_Pool <- rbind(RES_Pool, cbind(RES_List[[i]],
+                                       Set=rep(Set$Nom_ObjHC[i], nrow(RES_List[[i]]))))
+ }
> VAR_List <- c(VAR_List, as.list(RES_Pool))
```

3 Supervised analysis, STS vs. LTS

3.1 Merge of Bailey and TCGA sets, learning set

```
> require(inSilicoMerging)
> # Import Bailey and TCGA expression set object
> LearnObj <- list()
> load(paste(Path, "Rdata_ObjSets\\2016_Bailey_Nat2016_S4.RData", sep="\\"))
> LearnObj$Bailey <- Eset_Obj
> load(paste(Path, "Rdata_ObjSets\\20151020_PAAD_TCGA_RNAseqV2_S4.RData", sep="\\"))
> LearnObj$TCGA <- Eset_Obj
> rm(Eset_Obj)
> # Merge both set,
> Eset_Obj <- merge(LearnObj, method="COMBAT")  # merge w/ empirical Bayes normalisation
> Eset_None <- merge(LearnObj, method="NONE")   # merge w/o inter-set normalisation (ctrl)
```

3.2 Normalization evaluation with PCA and Bailey molecular subtypes

Objects preparation on Bailey's molecular subtypes genes

```
> # Bailey et al. genes list : obj. ref
> load("G:\\dat ext\\R.Ref.GES\\Bailey.Classifier.RData")
> # Prep. merge data object
> source("g:/Ori Tools/Prog/Rwork/Script/data handle/Manip.data.r")
> load(paste(Path, "Rdata_ObjSets\\chip", annotation(Eset_Obj), sep="\\"))  # load chip annot.
> CHIP <- CHIP[match(featureNames(Eset_Obj), CHIP$PID),]
> Obj_Bailey <- export.data.PF(exprs(Eset_Obj), CHIP$GeneID, ref, ref$GeneID)
> Obj_Bailey$INFO <- pData(Obj_Bailey)
> # Prep. merge data ctrl object
> load(paste(Path, "Rdata_ObjSets\\chip", annotation(Eset_None), sep="\\"))  # load chip annot.
> CHIP <- CHIP[match(featureNames(Eset_None), CHIP$PID),]
> Obj_Bailey_None <- export.data.PF(exprs(Eset_None), CHIP$GeneID, ref, ref$GeneID)
> Obj_Bailey_None$INFO <- pData(Obj_Bailey_None)
```

PCA preparation, selection of PACA and visualization groups

```
> selPrim <- which(Obj_Bailey$INFO$Type %in% "Primary" &
+                         Obj_Bailey$INFO$Duplicate %in% "unique" &
+                         Obj_Bailey$INFO$OS.Evt >= 0 & Obj_Bailey$INFO$OS.Del >= 0)
> # Grps defintion
> GRP_Subtype <- Obj_Bailey$INFO$Bailey
> GRP_SubtypeCol <- ifelse(GRP_Subtype %in% "ADEX", "brown",
+                          ifelse(GRP_Subtype %in% "Squamous", "blue",
+                                 ifelse(GRP_Subtype %in% "Immunogenic", "red",
+                                        ifelse(GRP_Subtype %in% "Pancreatic_Progenitor",
+                                               "orange", NA))))
> GRP_Set <- c(rep("ICGC-RNAseq",ncol(LearnObj$Bailey)), rep("TCGA-RNAseq",ncol(LearnObj$TCGA)))
> GRP_Setcol <- ifelse(GRP_Set == "ICGC-RNAseq", "blue", "red")
```

PCA w/ plots

```
> source("G:\\Ori Tools\\Prog\\Rwork\\Script\\PCA_AutoPlot.r")
> par(mfrow=c(2,2))
> # PCA ctrl none ~ set
> PCA_PF(DATA=Obj_Bailey_None$data.sel[,selPrim], Cex=0.5,
+        GRP=GRP_Set[selPrim], GRP_COL=GRP_Setcol[selPrim], Ellipse=TRUE, Traits=F, IC="CI95")
> legend("topleft", c("ICGC-RNAseq", "TCGA-RNAseq"), pch=19, col=c("blue", "red"),
+        box.col=0, cex=0.75)
> title("ICGC- & TCGA-RNAseq, Raw") ; box()
> # PCA ctrl none ~ subtypes
> PCA_PF(Obj_Bailey_None$data.sel[,selPrim], Cex=0.5,
+        GRP_Subtype[selPrim], GRP_SubtypeCol[selPrim], Ellipse=TRUE, Traits=F, IC="CI95")
> legend("topleft", c("ADEX", "Immunogenic", "Progenitor", "Squamous"), pch=19,
+        col=c("brown", "red", "orange", "blue"), box.col=0, cex=0.75)
> title("ICGC- & TCGA-RNAseq, Raw") ; box()
> # PCA norm. sets ~ set
> PCA_PF(Obj_Bailey$data.sel[,selPrim], Cex=0.5,
+        GRP_Set[selPrim], GRP_Setcol[selPrim], Ellipse=TRUE, Traits=F, IC="CI95")
> legend("bottomleft", c("ICGC-RNAseq", "TCGA-RNAseq"), pch=19,
+        col=c("blue", "red"), box.col=0, cex=0.75)
> title("ICGC- & TCGA-RNAseq, Norm.") ; box()
> # PCA norm. sets ~ subtypes
> PCA_PF(Obj_Bailey$data.sel[,selPrim], Cex=0.5,
+        GRP_Subtype[selPrim], GRP_SubtypeCol[selPrim], Ellipse=TRUE, Traits=F, IC="CI95")
> legend("bottomleft", c("ADEX", "Immunogenic", "Progenitor", "Squamous"), pch=19,
+        col=c("brown", "red", "orange", "blue"), box.col=0, cex=0.75)
> title("ICGC- & TCGA-RNAseq, Norm.") ; box()
```

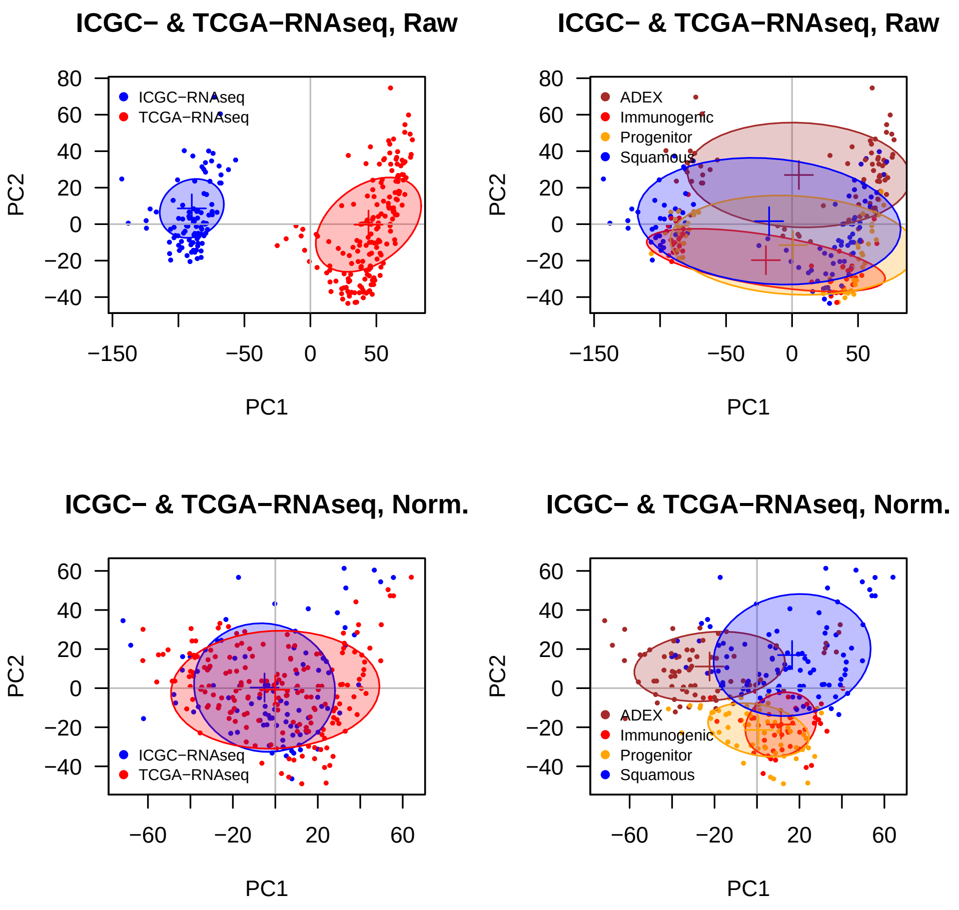

3.3 Supervised analysis, STS vs. LTS

3.3.1 Limma, moderated t-statistics w/ empirical Bayes shrinkage

```
> rm(Eset_None, CHIP)
> load(paste(Path, "Rdata_ObjSets\\chip", annotation(Eset_Obj), sep="\\"))  # chip annot.
> CHIP <- CHIP[match(featureNames(Eset_Obj), CHIP$PID),]
> GRP_DS <- rep(NA, ncol(Eset_Obj))
> GRP_DS[intersect(selPrim,
+                   which(pData(Eset_Obj)$OS.Evt == 1  & pData(Eset_Obj)$OS.Del <= 6))] <- "STS"
> GRP_DS[intersect(selPrim,
+                   which(pData(Eset_Obj)$OS.Evt == 0  & pData(Eset_Obj)$OS.Del >= 36))] <- "LTS"
> source("G:\\Ori Tools\\Prog\\Rwork\\Script\\20161017_MEF_Limma_fitoutput_optPaired.r")
> # Limma, moderated t-statistics of differential expression by empirical Bayes shrinkage
> DS_STS6m36m <- fitLimmaeBayes_PF(exprs(Eset_Obj), GRP=GRP_DS)
> # Selection of significant genes following chosen thresholds (i.e. p<5%, q<25% & |FC|>2x)
> DS_STS6m36m <- LimmaSignificant_Genes(DS_STS6m36m,
+                                      Threshold=list(p=0.05, q=0.05, FC=1))  # FC : log2
```

Volcano plot of the analysis

```
> par(mfrow=c(1,1))
> VolcPlot_LimmaRes(DS_STS6m36m)
> text(c(3,-3), y=c(7,7), paste("N =", unlist(lapply(DS_STS6m36m$selGeneDS,length))),
+      col=c("red", "green3"), cex=0.85)
> title(paste("moderated t-test, limma\n", length(which(GRP_DS %in% "STS")),
+             "STS vs.", length(which(GRP_DS %in% "LTS")), "LTS"))
```

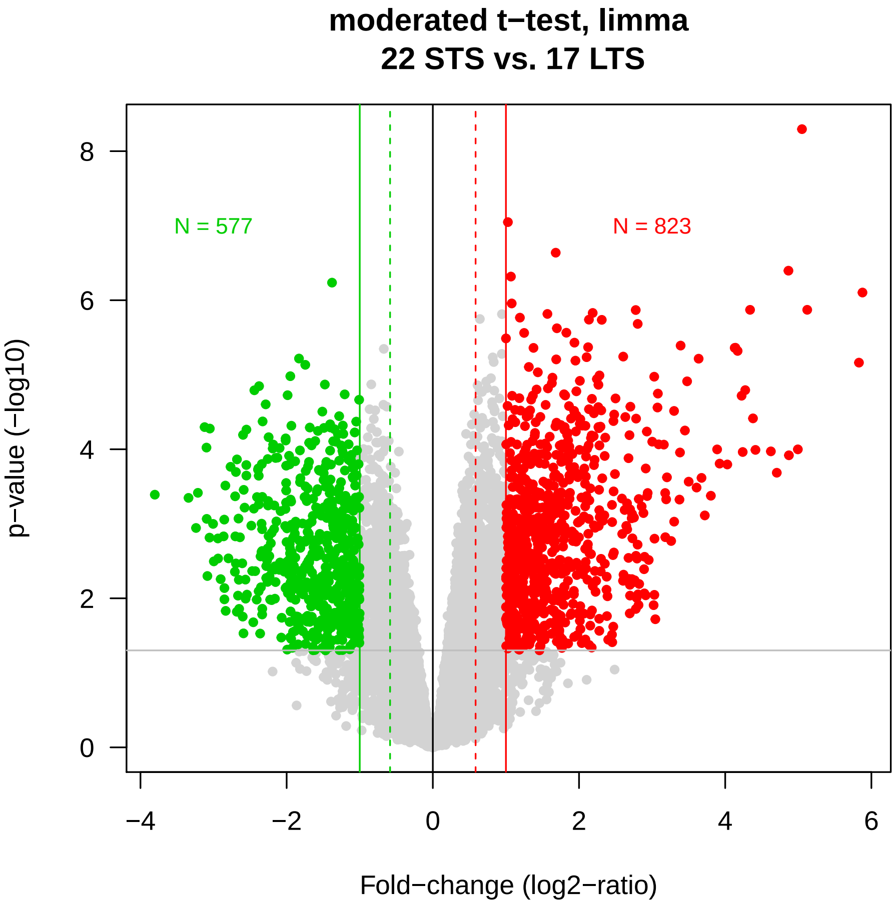

3.3.2 LASSO selection from 1400 genes

```
> require(glmnet)
> sel_STS636 <- intersect(selPrim, which(!is.na(GRP_DS)))
> Mat_GES <- as.matrix(t(exprs(Eset_Obj)[
+       match(DS_STS6m36m$RES$GeneID[unlist(DS_STS6m36m$selGeneDS)], featureNames(Eset_Obj)),
+       sel_STS636]))
> fit_regL <- glmnet(Mat_GES, GRP_DS[sel_STS636], family="binomial", alpha=1)
> fit_regL_CVdev <- cv.glmnet(Mat_GES, GRP_DS[sel_STS636], family="binomial",
+                             type.measure="deviance", nfolds=1e2)
> fit_regL_coef <- coef(fit_regL, s=fit_regL_CVdev$lambda.min)
> # Plot Cross-Validation LASSO model
> par(mfrow=c(1,2))
> plot(fit_regL_CVdev,las=1, main="Lasso fit CV")
> abline(v=log(fit_regL_CVdev$lambda.min), col="orange", lwd=2)
> text(log(fit_regL_CVdev$lambda.min), 1.4, paste("lambda.min=",
+                                         round(fit_regL_CVdev$lambda.min,4),"\n",
+                                         length(fit_regL_coef@i)-1, " genes" ,sep=""),
+      col="orange", cex=0.75, pos=4)
> # Plot lambda fit
> plot(fit_regL, xvar="lambda",las=1, main="Lasso coefficient")
> abline(v=log(fit_regL_CVdev$lambda.min), col="orange", lwd=2)
```

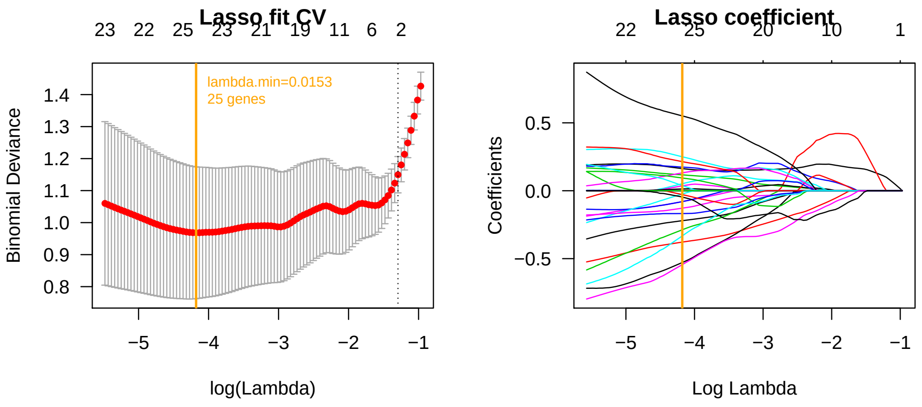

3.3.3 25-genes model classification preparation

```
> Model <- list(data=exprs(Eset_Obj)[
+              match(rownames(fit_regL_coef)[fit_regL_coef@i[!fit_regL_coef@i == 0]+1],
+                    featureNames(Eset_Obj)),],
+            ref=CHIP[
+              match(rownames(fit_regL_coef)[fit_regL_coef@i[!fit_regL_coef@i == 0]+1],
+                    CHIP$PID),],
+            grp=GRP_DS,
+            subset=selPrim)
> Model$ref$coef <- fit_regL_coef[fit_regL_coef@i[!fit_regL_coef@i == 0]+1]
> print(xtable(Model$ref[,c(3,5,6,9)],caption="25-genes model", table.placement="!h"),
+       size="footnotesize", include.rownames = FALSE)
```

Gene.Symbol	map_location	EntrezGeneID	coef
GPR87	3q24	53836	0.16
KRT13	17q21.2	3860	0.12
RAC2	22q13.1	5880	0.17
C16orf74	16q24.1	404550	0.05
NAMPT	7q22.3	10135	0.04
DHRS9	2q31.1	10170	0.01
HIST2H2BF	1q21.2	440689	0.25
TREM2	6p21.1	54209	0.13
ZDHHC20	13q12.11	253832	0.55
CD180	5q12	4064	0.21
ADGRG6	6q24.1	57211	0.09
APBB1IP	10p12.1	54518	0.04
EGR3	8p23-p21	1960	-0.08
MACROD2	20p12.1	140733	-0.22
EPHA7	6q16.1	2045	-0.38
RASGEF1A	10q11.21	221002	-0.29
SYNM	15q26.3	23336	-0.08
S100A1	1q21	6271	-0.02
WNK2	9q22.3	65268	-0.13
RAMP2	17q12-q21.1	10266	-0.53
SOCS2	12q	8835	-0.03
COL28A1	7p21.3	340267	-0.17
B4GALT6	18q11	9331	-0.33
PLCB4	20p12	5332	-0.54
MTURN	7p14.3	222166	-0.05

25-genes model

4 25-genes model, public data

4.1 Batch classification

```
> rm(Eset_Obj,CHIP)
> RES_List <- list()
> for ( i in 1:nrow(Set)){
+   load(paste(Path, "RData_ObjSets", Set$FileName[i], sep="\\"))
+   #### import chip annot
+   Annotation <- annotation(Eset_Obj)
+   load(paste(Path, "RData_ObjSets\\chip", Annotation, ".RData", sep=""))
+   CHIP <- CHIP[match(featureNames(Eset_Obj), CHIP$PID),]  # internal ctrl
+   #### data matrix
+   DATA <- exprs(Eset_Obj)
+   #### Classification :
+   ### 25-g model,
+   source("g:/Ori Tools/Prog/Rwork/Script/Classifier/20170410_Classif_GESsurvXtremRNAseq.r")
+   GES_PAADXtrem6m36m <- Classif_Bailey_PAAD_surv(DATA=DATA,
+                                               DATA_ID=CHIP$GeneID,
+                                               Model_Obj=Model,
+                                               Subset=Model$selPrim,
+                                               Nom=Set$Nom_ObjHC[i])
+         Tmp_25 <- data.frame(GES_PAADXtrem6m36m)
+         colnames(Tmp_25) <- paste("ges25g", colnames(Tmp_25), sep="_")
+         ###
+   rm(Eset_Obj, DATA, CHIP)
+ }
> #### Merge classifications
> RES_Pool <- c()
> for (i in 1:length(RES_List)){
+     RES_Pool <- rbind(RES_Pool, cbind(RES_List[[i]],
+                                 Set=rep(Set$Nom_ObjHC[i], nrow(RES_List[[i]]))))
+ }
> # final output
> GES25g <- RES_Pool$ges25g_grp_pred
```

Evaluation classification 25g-model, learning set pre-normalization :

```
> # Contingence table, observed vs. predicted groups
> print(xtable(table(Predicted=GES25g[selLearn], Observed=GRP_STS6m36m[selLearn]),
+ caption="25-genes model", table.placement="!h"),
+       size="small", include.rownames = TRUE)
```

	LTS	STS
LTS-like	17	0
STS-like	0	22

25-genes model

```
> # Overall survival (OS), Kaplan-Meier
> source("G:\\Ori Tools\\Prog\\Rwork\\Script\\survie.PF\\Survival.PF\\R\\Surv.PF-20160325.r")
> source("G:\\Ori Tools\\Prog\\Rwork\\Script\\survie.PF\\Survival.PF\\R\\PlotSurv.PF.R")
> KM_OS_25g_Learn <- Surv.PF(GES25g, VAR_ListSurv$OS$Evt, VAR_ListSurv$OS$Del, "Overall",
+                           Subset=selLearn, Y=24, Print = FALSE)
> PlotSurv.PF(KM_OS_25g_Learn, style=1, color=c(1,2), mark=FALSE, limit=60,
+             nom="25-gene model, learning", position=c(5,0.1), cex=0.8)
```

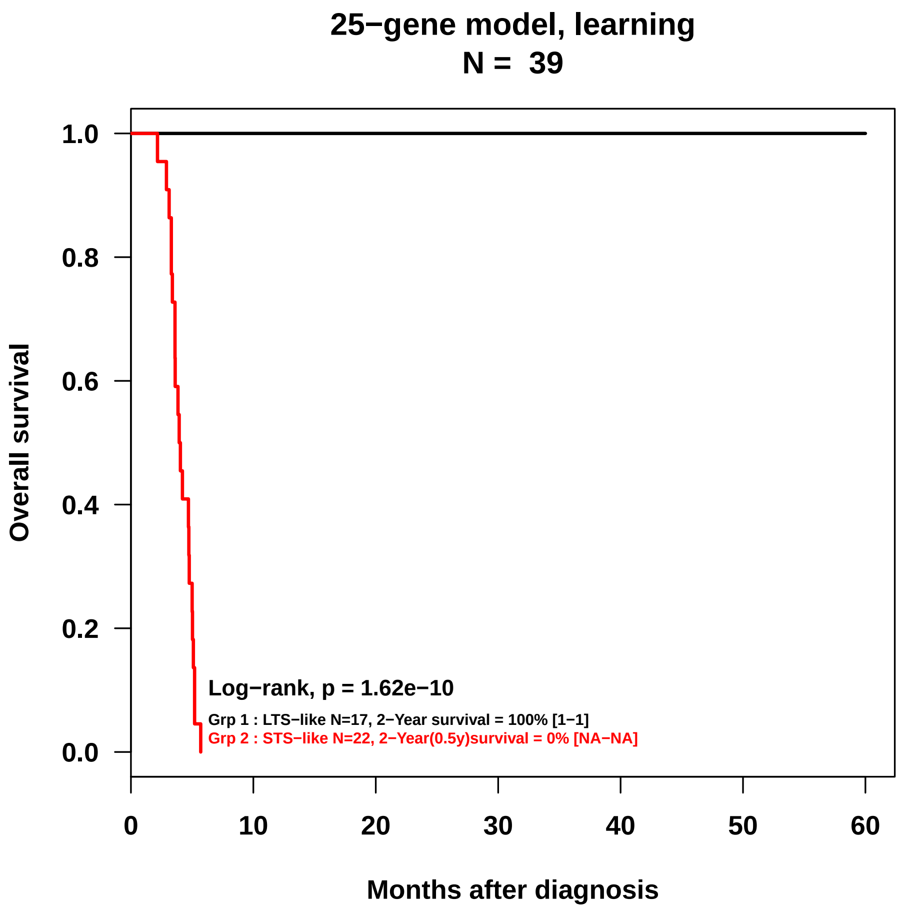

4.2 Validation set, statistics on 25-gene model (N=562)

4.2.1 Description groups of the 25-genes classification

```
> source("g:/Ori Tools/Prog/Rwork/Script/Table.PF/Table.PF.r")
> TabDesc_GES25g_valid <- Table_PF(GES25g, VAR_List, Subset=sel562)
> colnames(TabDesc_GES25g_valid)[c(3,6)] <- c("N", "p-value")
> print(xtable(TabDesc_GES25g_valid[,1:6],
+              caption="Association w/ 25-genes model groups", table.placement="!h"),
+       size="footnotesize", include.rownames = FALSE)
```

```
> KM_OS_Valid <- Surv.PF(NULL, VAR_ListSurv$OS$Evt, VAR_ListSurv$OS$Del,
+                        "Overall", Subset=sel562, Y=24, Print = FALSE)
> KM_OS_25g_Valid <- Surv.PF(GES25g, VAR_ListSurv$OS$Evt, VAR_ListSurv$OS$Del,
+                        "Overall", Subset=sel562, Y=24, Print = FALSE)
> par(mfrow=c(1,2))
> PlotSurv.PF(KM_OS_Valid, style=1, color=c(1), mark=FALSE, limit=60, pVal=FALSE,
+             nom="Validation set", position=c(1,0.1), cex=0.75)
> PlotSurv.PF(KM_OS_25g_Valid, style=1, color=c(1,2), mark=FALSE, limit=60,
+             nom="25-gene model, validation set", position=c(1,0.1), cex=0.75)
```

Var	Mod	N	LTS.like	STS.like	p-value
Age					0.219
	<=60	108	64(31%)	44(38%)	
	>60	217	145(69%)	72(62%)	
Sex					0.419
	female	157	105(50%)	52(44%)	
	male	171	106(50%)	65(56%)	
Stade					0.759
	1	54	35(12%)	19(11%)	
	2	403	255(84%)	148(85%)	
	3	10	5(2%)	5(3%)	
	4	11	8(3%)	3(2%)	
Histo					0.0868
	ductal	504	308(98%)	196(100%)	
	other	6	6(2%)	0(0%)	
Grade					0.00157
	1	27	24(16%)	3(3%)	
	2	138	90(59%)	48(54%)	
	3	75	38(25%)	37(42%)	
	4	2	1(1%)	1(1%)	
pT					0.879
	1	15	9(4%)	6(5%)	
	2	57	36(16%)	21(16%)	
	3	281	181(78%)	100(76%)	
	4	11	6(3%)	5(4%)	
pN					0.824
	0	123	79(30%)	44(29%)	
	1	291	183(70%)	108(71%)	
Collisson					1e-06
	Centroid_Classical.PDA	223	130(38%)	93(43%)	
	Centroid_Exocrine.like.PDA	194	147(42%)	47(22%)	
	Centroid_QM.PDA	145	69(20%)	76(35%)	
Moffitt_NMF_type					7.8e-15
	Basal_like	214	88(25%)	126(58%)	
	Classical	348	258(75%)	90(42%)	
Bailey					1e-06
	ADEX	128	114(33%)	14(6%)	
	Immunogenic	101	69(20%)	32(15%)	
	Pancreatic_Progenitor	133	90(26%)	43(20%)	
	Squamous	200	73(21%)	127(59%)	

Association w/ 25-genes model groups

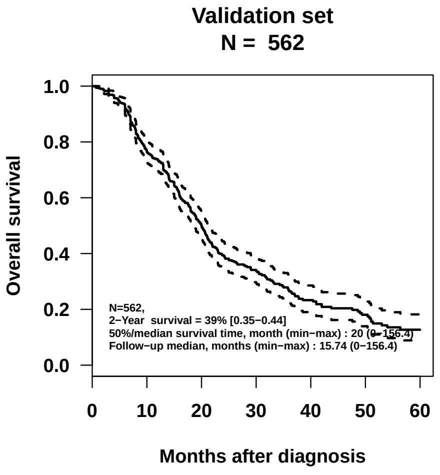

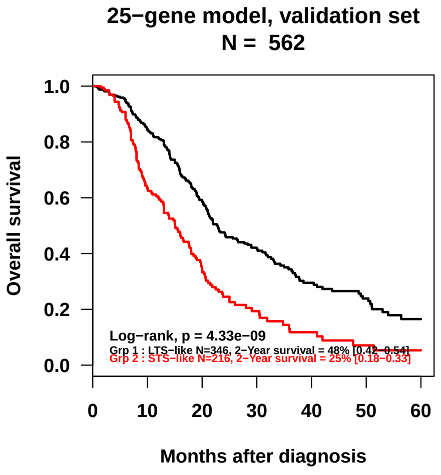

4.2.2 Univariate and multivariate OS analysis (valid, N=562)

Univariate all variables HC and 25-gene classification (Cox's regression)

```
> source("G:\\Ori Tools\\Prog\\Rwork\\Script\\20161108_UV.batch.r")
> UV_OS_Valid <- Batch.UV.PF(c(VAR_List, list(GES25g=GES25g)),
+                            VAR_ListSurv$OS$Evt, VAR_ListSurv$OS$Del,
+                            nom= "", Subset=sel562, PRINT=FALSE )
> print(xtable(UV_OS_Valid$Res.Batch[,c(1:4,6)],
+              caption="Univariate on validation set",
+              table.placement="!h"),
+       size="footnotesize", include.rownames = FALSE)
```

VAR	MODTEST	n	HR.95CI.	p
Age	>60	325	1.22 [0.88-1.7]	0.234
Sex	male	328	1.08 [0.8-1.45]	0.633
Stade	2	478	2.01 [1.32-3.07]	4.71e-03
	3		3.11 [1.33-7.23]	
	4		2.85 [1.16-7.05]	
Histo	other	510	0.36 [0.09-1.45]	0.151
Grade	2	242	1.52 [0.65- 3.55]	0.185
	3		2.15 [0.91- 5.11]	
	4		2.66 [0.53-13.29]	
pT	2	364	1.49 [0.62-3.59]	0.131
	3		1.95 [0.86-4.42]	
	4		2.93 [1.01-8.48]	
pN	1	414	1.83 [1.34-2.48]	1.24e-04
Collisson	Centroid_Exocrine.like.PDA	562	1.00 [0.77-1.29]	2.32e-03
	Centroid_QM.PDA		1.52 [1.17-1.99]	
Moffitt_NMF_type	Classical	562	0.64 [0.51-0.8]	6.29e-05
Bailey	Immunogenic	562	0.81 [0.57-1.17]	8.98e-06
	Pancreatic_Progenitor		0.97 [0.70-1.35]	
	Squamous		1.64 [1.22-2.19]	
GES25g	STS-like	562	1.93 [1.55-2.42]	7.47e-09

Univariate on validation set

Multivariate significant variables HC and 25-gene classification (Cox's regression)

```
> MV_OS_HC_Valid <- MV.PF(coxph(Surv(VAR_ListSurv$OS$Del, VAR_ListSurv$OS$Evt) ~
+                               Stade + pN + GES25g, data=VAR_List, subset=sel562))
> print(xtable(MV_OS_HC_Valid$Cox.MV,
+              caption="Multivariate, histo-clinical + 25-gene",
+              table.placement="!h"),
+       size="small", include.rownames = TRUE)
```

	N	HR	CI95	p
Stade2	408	1.57	[0.88-2.82]	0.128
Stade3	408	2.21	[0.82-5.97]	0.119
Stade4	408	1.44	[0.19-10.97]	0.723
pN1	408	1.5	[1.04-2.16]	2.95e-02
GES25gSTS-like	408	2.04	[1.54-2.7]	6.33e-07

Multivariate, histo-clinical + 25-gene

Multivariate significant variables HC and 25-gene classification (Cox's regression)

```
> MV_OS_Subtype_Valid <- MV.PF(coxph(Surv(VAR_ListSurv$OS$Del, VAR_ListSurv$OS$Evt) ~
+                               Collisson + Moffitt_NMF_type + Bailey + GES25g,
+                            data=VAR_List, subset=sel562))
> print(xtable(MV_OS_Subtype_Valid$Cox.MV,
+              caption="Multivariate, molecular subtypes + 25-gene",
+              table.placement="!h"),
+       size="small", include.rownames = TRUE)
```

	N	HR	CI95	p
CollissonCentroid_Exocrine.like.PDA	562	0.94	[0.66-1.34]	0.732
CollissonCentroid_QM.PDA	562	1.15	[0.83-1.59]	0.395
Moffitt_NMF_typeClassical	562	1	[0.72-1.38]	0.994
BaileyImmunogenic	562	0.68	[0.43-1.06]	0.09
BaileyPancreatic_Progenitor	562	0.79	[0.51-1.23]	0.302
BaileySquamous	562	1.09	[0.68-1.74]	0.731
GES25gSTS-like	562	1.77	[1.38-2.26]	6.33e-06

Multivariate, molecular subtypes + 25-gene

4.3 Evaluation 25-gene model and molecular subtypes

```
> # Surv. objects
> KM_OS_25g_Collisson <- Surv.PF(interaction(GES25g, VAR_List$Collisson),
+                               VAR_ListSurv$OS$Evt, VAR_ListSurv$OS$Del,
+                               nom= "Overall", Subset=sel562, Print=FALSE, Y=24)
> KM_OS_25g_Moffitt_type <- Surv.PF(interaction(GES25g, VAR_List$Moffitt_NMF_type),
+                               VAR_ListSurv$OS$Evt, VAR_ListSurv$OS$Del,
+                               nom= "Overall", Subset=sel562, Print=FALSE, Y=24)
> KM_OS_25g_Bailey <- Surv.PF(interaction(GES25g, VAR_List$Bailey),
+                            VAR_ListSurv$OS$Evt, VAR_ListSurv$OS$Del,
+                            nom= "Overall", Subset=sel562, Print=FALSE, Y=24)
> # Kaplan-Meier plot, surv objects
> par(mfrow=c(2,2))
> # Collisson + 25-gene
> PlotSurv.PF(KM_OS_25g_Collisson, style=rep(c(2,1),3),
+             color=c(rep("orange",2), rep("blue",2), rep("red",2)),
+             nom="Collisson + GES25g", mark=F, LWD=rep(c(1,3),3), limit=60,
+             position=c(0,0.1), pVal=TRUE, texte=FALSE, cex=0.8)
> legend(0,0.4,c("STS-like", "LTS-like"), box.col=0, lty=c(1,2), lwd=c(3,1), cex=0.5)
> legend("topright", c("Exocrine-like", "Classical", "Quasi-mesenchymal"),
+        text.col=c("blue", "orange", "red"), cex=0.75, box.col=0) ; box()
> # Moffitt, tumor + 25-gene
> PlotSurv.PF(KM_OS_25g_Moffitt_type, style=rep(c(2,1),2),
+             color=c(rep("red",2), rep("orange",2)),
+             nom="Mofffitt tumor + GES25g", mark=F, LWD=rep(c(1,3),2), limit=60,
+             position=c(0,0.1), pVal=TRUE, texte=FALSE, cex=0.8)
> legend(0,0.4,c("STS-like", "LTS-like"), box.col=0, lty=c(1,2), lwd=c(3,1), cex=0.5)
> legend("topright", c("Classical", "Basal"),
+        text.col=c("orange", "red"), cex=0.75, box.col=0) ; box()
> # Bailey + 25-gene
> PlotSurv.PF(KM_OS_25g_Bailey, style=rep(c(2,1),4),
+             color=c(rep("blue",2), rep("green3",2), rep("orange",2), rep("red",2)),
+             nom="Bailey + GES25g", mark=F, LWD=rep(c(1,3),4), limit=60,
+             position=c(0,0.1), pVal=TRUE, texte=FALSE, cex=0.8)
> legend(0,0.4,c("STS-like", "LTS-like"), box.col=0, lty=c(1,2), lwd=c(3,1), cex=0.5)
> legend("topright", c("Pancreatic progenitor", "Immunogenic", "Squamous", "ADEX"),
+        text.col=c("orange", "green3", "red", "blue"), cex=0.75, box.col=0) ; box()
```

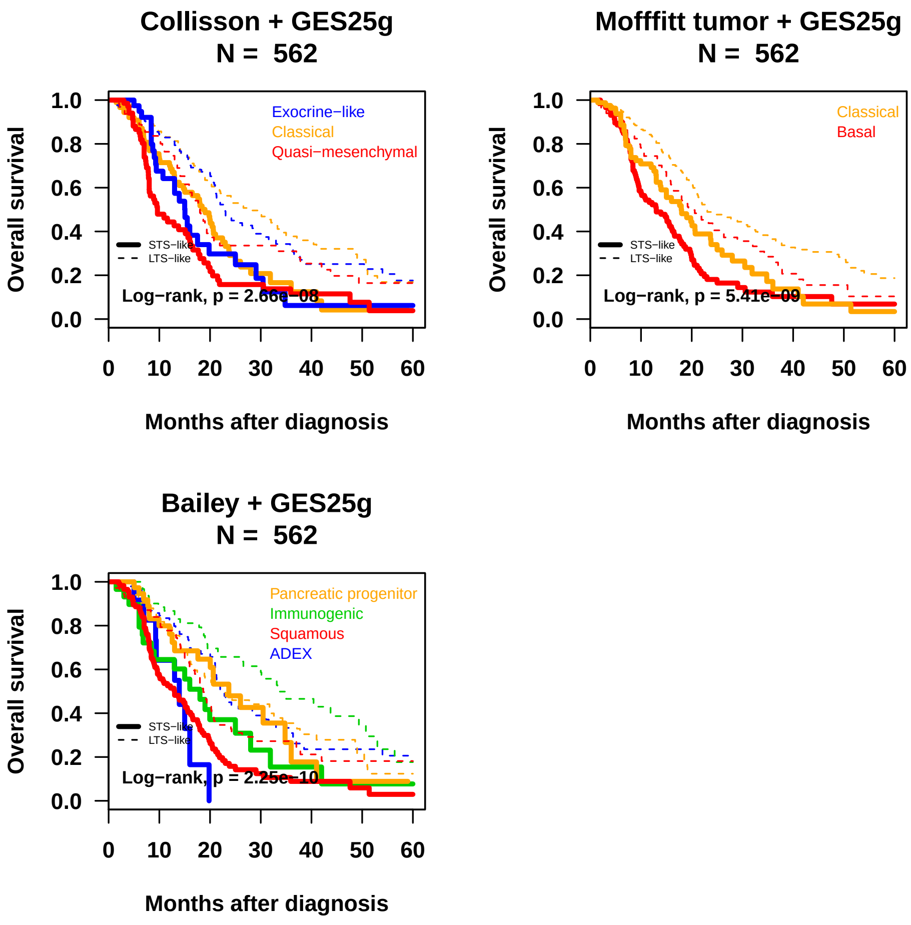

Kaplan-Meier curves of molecular subtypes w/ 25-gene classification
