# Supplementary material for: A 25-gene classifier predicts overall survival in resectable pancreatic cancer
Source: BMC Med. 2017 Sep 20;15:170. doi: 10.1186/s12916-017-0936-z (PMC5606023; doi:10.1186/s12916-017-0936-z)
Supplement: Supplementary file 6 — Gene overlap between our 25-gene signature and other prognostic signatures. Venn diagram showing the overlap in genes between our signature and three prognostic signatures (A, Wang’s 28-gene signature, Haider’s 36-gene signature, and Chen’s 15-gene signature; the Stratford’s 6-gene and the Kirby’s 19-gene signatures are not shown because they display no gene common with the other four signatures), and between our signature and the four molecular subtype classifiers (B, Bailey’s 859-gene classifier, Collisson’s 62-gene classifier, Moffitt’s tumor 50-gene classifier, and Moffitt’s stroma 48-gene classifier). (PPTX 131 kb) [file 12916_2017_936_MOESM6_ESM.pptx]

## Slide 1
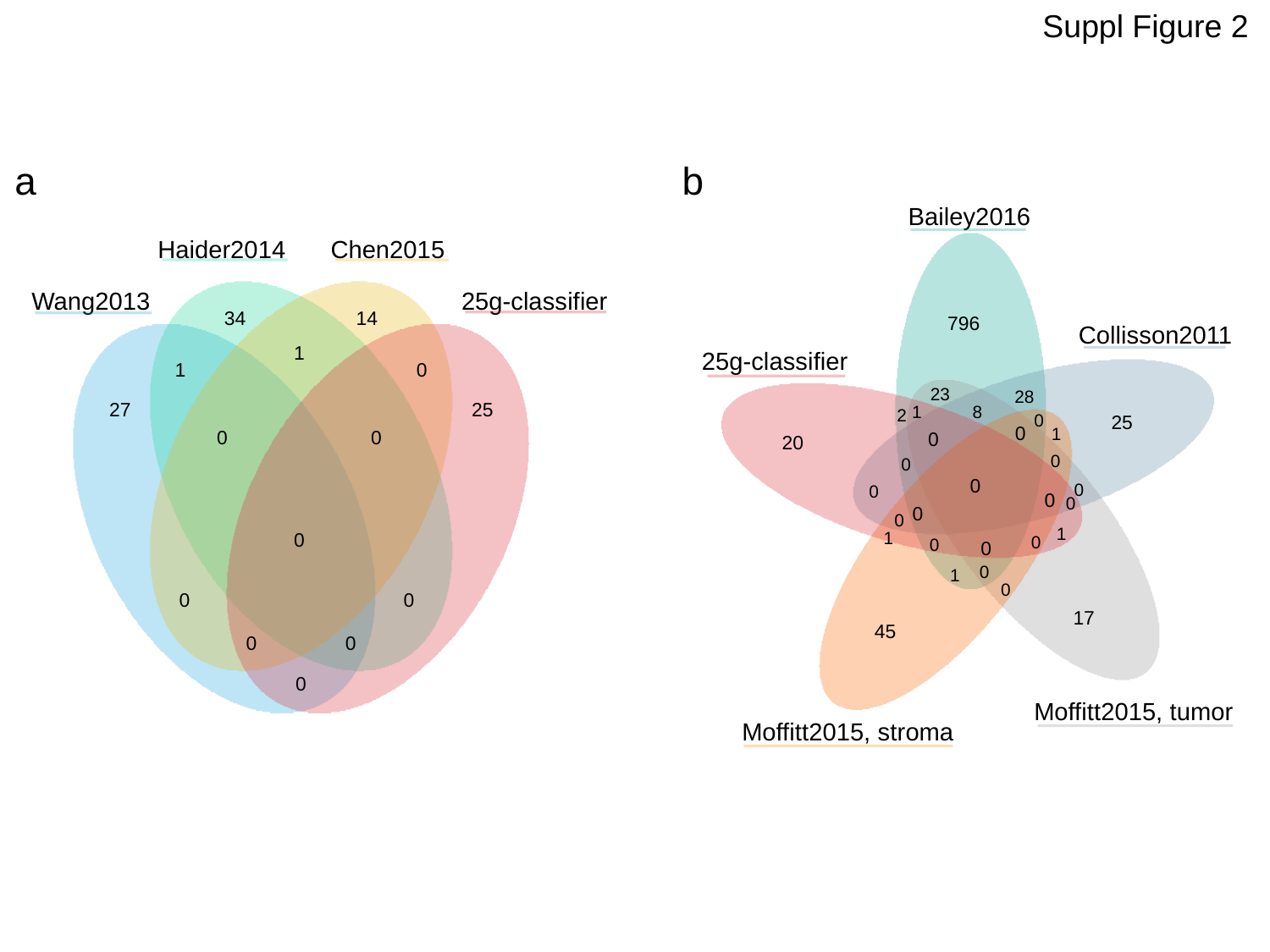

Suppl Figure 2
a
b
Bailey2016
796
Collisson2011
25g-classifier
23
28
8
1
2
0
25
0
1
0
20
0
0
0
0
0
0
0
0
0
1
1
0
0
0
0
1
0
17
45
Moffitt2015, tumor
Moffitt2015, stroma
Haider2014
Chen2015
Wang2013
25g-classifier
34
14
1
1
0
27
25
0
0
0
0
0
0
0
0
