# Supplementary material for: A 25-gene classifier predicts overall survival in resectable pancreatic cancer
Source: BMC Med. 2017 Sep 20;15:170. doi: 10.1186/s12916-017-0936-z (PMC5606023; doi:10.1186/s12916-017-0936-z)
Supplement: Supplementary file 8 — Overall survival (OS) in each set of the pooled validation set according to our prognostic 25-gene classifier. Kaplan–Meier OS curves in all patients according to our prognostic classifier (STS-like and LTS-like classes). The dashed vertical line represents the 2-year OS. The P values of the log-rank test are indicated. (PPTX 142 kb) [file 12916_2017_936_MOESM8_ESM.pptx]

## Slide 1
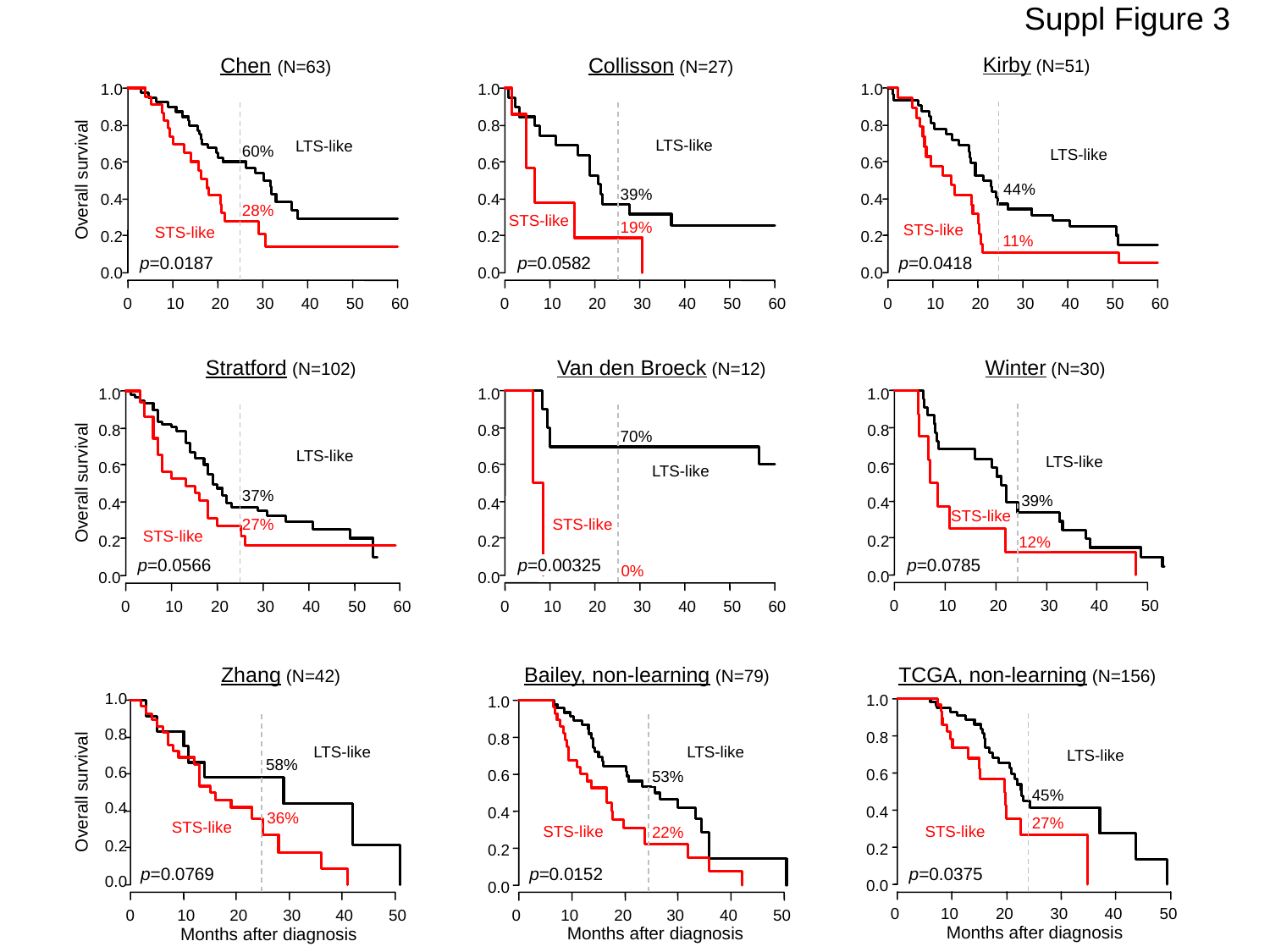

Suppl Figure 3
Kirby (N=51)
Chen (N=63)
Collisson (N=27)
1.0
1.0
1.0
0.8
0.8
0.8
LTS-like
LTS-like
60%
LTS-like
0.6
0.6
0.6
Overall survival
44%
39%
0.4
0.4
0.4
28%
STS-like
19%
STS-like
STS-like
0.2
0.2
0.2
11%
p=0.0187
p=0.0582
p=0.0418
0.0
0.0
0.0
0
10
20
30
40
50
60
0
10
20
30
40
50
60
0
10
20
30
40
50
60
Winter (N=30)
Van den Broeck (N=12)
Stratford (N=102)
1.0
1.0
1.0
0.8
0.8
0.8
70%
LTS-like
LTS-like
0.6
0.6
0.6
LTS-like
Overall survival
37%
39%
0.4
0.4
0.4
STS-like
STS-like
27%
STS-like
0.2
0.2
0.2
12%
p=0.0566
p=0.00325
p=0.0785
0%
0.0
0.0
0.0
0
10
20
30
40
50
0
10
20
30
40
50
60
0
10
20
30
40
50
60
Zhang (N=42)
Bailey, non-learning (N=79)
TCGA, non-learning (N=156)
1.0
1.0
1.0
0.8
0.8
0.8
LTS-like
LTS-like
LTS-like
58%
0.6
0.6
0.6
53%
Overall survival
45%
0.4
0.4
0.4
36%
27%
STS-like
STS-like
STS-like
22%
0.2
0.2
0.2
p=0.0769
p=0.0152
p=0.0375
0.0
0.0
0.0
0
10
20
30
40
50
0
10
20
30
40
50
0
10
20
30
40
50
Months after diagnosis
Months after diagnosis
Months after diagnosis
