# Supplementary material for: A 25-gene classifier predicts overall survival in resectable pancreatic cancer
Source: BMC Med. 2017 Sep 20;15:170. doi: 10.1186/s12916-017-0936-z (PMC5606023; doi:10.1186/s12916-017-0936-z)
Supplement: Supplementary file 9 — Overall survival (OS) in the validation set according to our prognostic 25-gene classifier and the American Joint Committee on Cancer (AJCC) Tumor, Node and Metastasis stage. Kaplan–Meier OS curves according to our prognostic classifier (STS-like and LTS-like classes) in patients with AJCC stage 1 (a) and AJCC stage 2 (b). The P values of the log-rank test are indicated. (PPTX 78 kb) [file 12916_2017_936_MOESM9_ESM.pptx]

## Slide 1
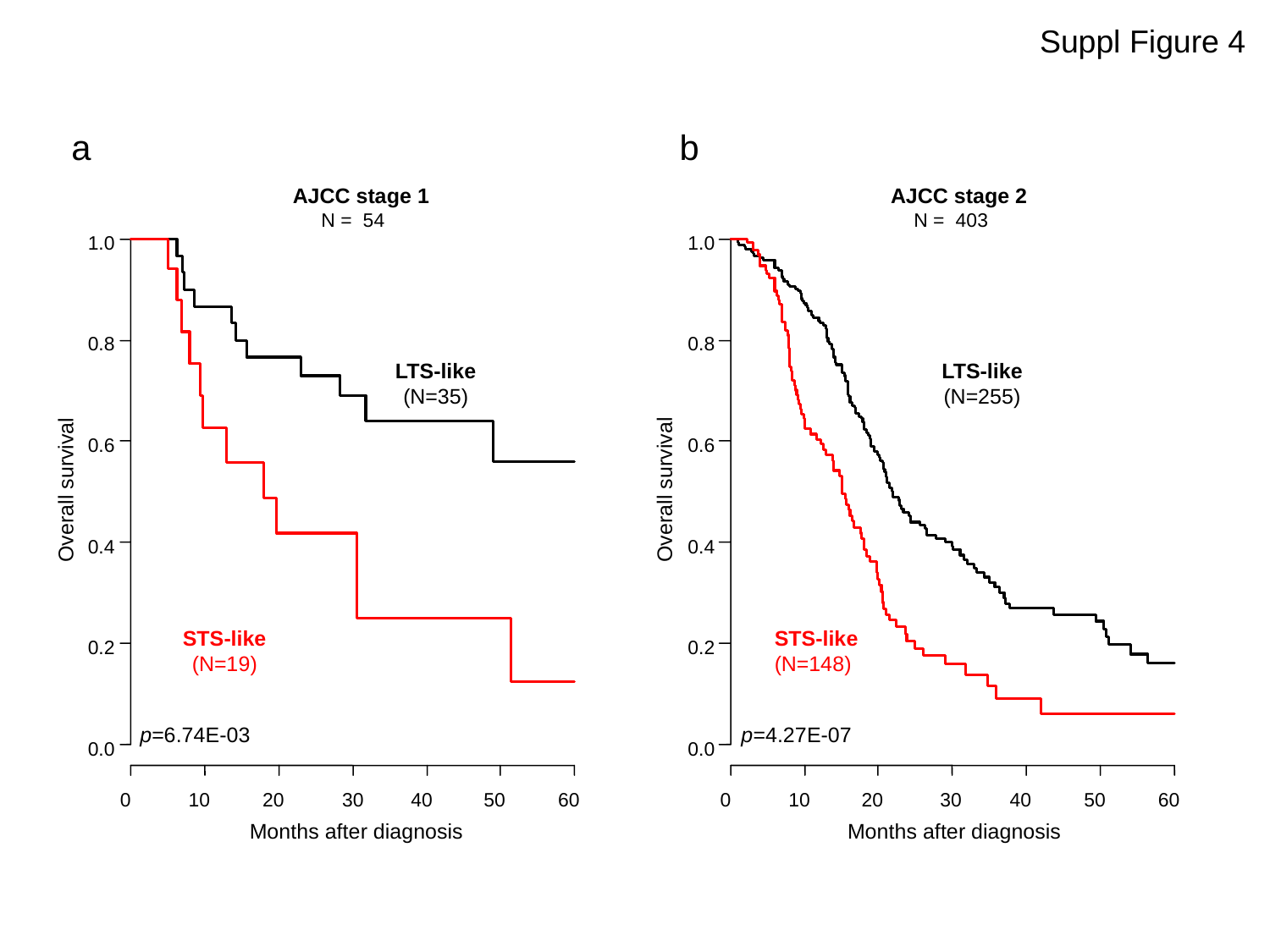

Suppl Figure 4
a
b
AJCC stage 1
AJCC stage 2
N = 54
N = 403
1.0
1.0
0.8
0.8
LTS-like
(N=35)
LTS-like
(N=255)
0.6
0.6
Overall survival
Overall survival
0.4
0.4
STS-like
(N=19)
STS-like
(N=148)
0.2
0.2
p=6.74E-03
p=4.27E-07
0.0
0.0
0
10
20
30
40
50
60
0
10
20
30
40
50
60
Months after diagnosis
Months after diagnosis
