# Supplementary material for: A 25-gene classifier predicts overall survival in resectable pancreatic cancer
Source: BMC Med. 2017 Sep 20;15:170. doi: 10.1186/s12916-017-0936-z (PMC5606023; doi:10.1186/s12916-017-0936-z)
Supplement: Supplementary file 10 — Overall survival (OS) in the validation set according to the molecular subtypes. Kaplan–Meier OS curves according to the molecular subtypes defined by Bailey (a), Collison (b), and Moffitt (c). The P values of the log-rank test are indicated. (PPTX 103 kb) [file 12916_2017_936_MOESM10_ESM.pptx]

## Slide 1
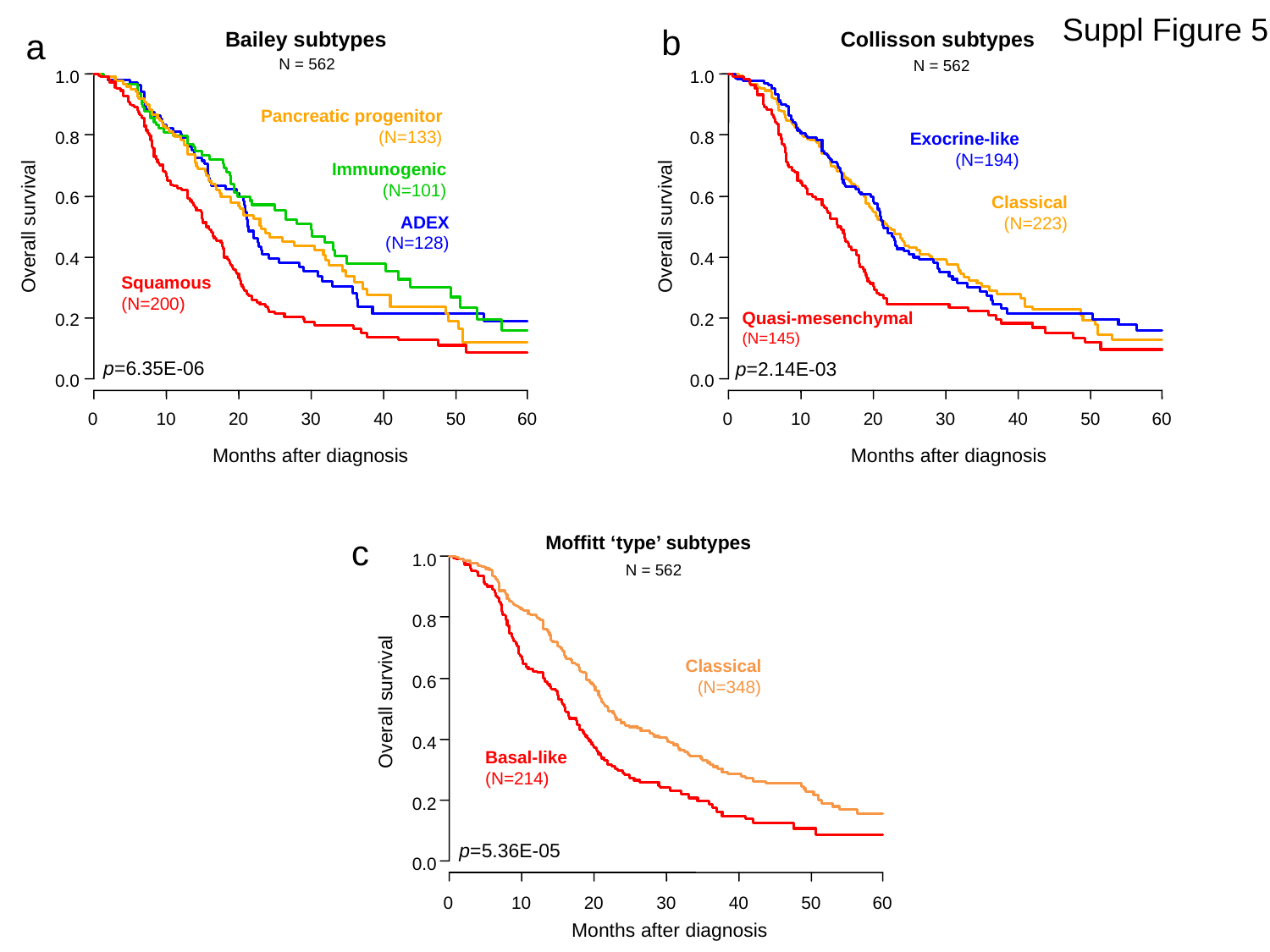

Suppl Figure 5
b
a
Bailey subtypes
N = 562
1.0
Pancreatic progenitor
(N=133)
0.8
Immunogenic
(N=101)
0.6
ADEX
(N=128)
Overall survival
0.4
Squamous
(N=200)
0.2
p=6.35E-06
0.0
0
10
20
30
40
50
60
Months after diagnosis
Collisson subtypes
N = 562
1.0
0.8
Exocrine-like
(N=194)
0.6
Classical
(N=223)
Overall survival
0.4
Quasi-mesenchymal
(N=145)
0.2
p=2.14E-03
0.0
0
10
20
30
40
50
60
Months after diagnosis
c
Moffitt ‘type’ subtypes
1.0
N = 562
0.8
Classical
(N=348)
0.6
Overall survival
0.4
Basal-like
(N=214)
0.2
p=5.36E-05
0.0
0
10
20
30
40
50
60
Months after diagnosis
